# Geospatial Science and Point-of-Care Testing: Creating Solutions for Population Access, Emergencies, Outbreaks, and Disasters

**DOI:** 10.3389/fpubh.2019.00329

**Published:** 2019-11-26

**Authors:** Gerald J. Kost

**Affiliations:** ^1^Point-of-Care Testing Center for Teaching and Research (POCT·CTR™), University of California, Davis, Davis, CA, United States; ^2^Knowledge Optimization^®^, Davis, CA, United States

**Keywords:** Demographic care unit, emergency management and preparedness, disasters, epidemics, geospatial care path™, point-of-care testing, small-world network, spatial care path™

## Abstract

**Objectives:** (a) To understand how to integrate geospatial concepts when implementing point-of-care testing (POCT); (b) to facilitate emergency, outbreak, and disaster preparedness and emergency management in healthcare small-world networks; (c) to enhance community resilience by using POCT in tandem with geographic information systems (GISs) and other geospatial tools; and (d) to advance crisis standards of care at points of need, adaptable and scalable for public health practice in limited-resource countries and other global settings.

**Content:** Visual logistics help integrate and synthesize POCT and geospatial concepts. The resulting geospatial solutions presented here comprise: (1) small-world networks and regional topography; (2) space-time transformation, hubs, and asset mapping; (3) spatial and geospatial care paths™; (4) GIS-POCT; (5) isolation laboratories, diagnostics isolators, and mobile laboratories for highly infectious diseases; (6) alternate care facilities; (7) roaming POCT—airborne, ambulances, space, and wearables; (8) connected and wireless POCT outside hospitals; (9) unmanned aerial vehicles; (10) geospatial practice—demographic care unit resource scoring, geographic risk assessment, and national POCT policy and guidelines; (11) the hybrid laboratory; and (12) point-of-careology.

**Value:** Small-world networks and their connectivity facilitate efficient and effective placement of POCT for optimal response, rescue, diagnosis, and treatment. Spatial care paths™ speed transport from primary encounters to referral centers bypassing topographic bottlenecks, process gaps, and time-consuming interruptions. Regional GISs position POCT close to where patients live to facilitate rapid triage, decrease therapeutic turnaround time, and conserve economic resources. Geospatial care paths™ encompass demographic and population access features. Timeliness creates value during acute illness, complex crises, and unexpected disasters. Isolation laboratories equipped with POCT help stop outbreaks and safely support critically ill patients with highly infectious diseases. POCT-enabled spatial grids can map sentinel cases and establish geographic limits of epidemics for ring vaccination.

**Impact:** Geospatial solutions generate inherently optimal and logical placement of POCT conceptually, physically, and temporally as a means to improve crisis response and spatial resilience. If public health professionals, geospatial scientists, and POCT specialists join forces, new collaborative teamwork can create faster response and higher impact during disasters, complex crises, outbreaks, and epidemics, as well as more efficient primary, urgent, and emergency community care.

## Goal and Mission

The goal of this review is to analyze the geospatial point-of-care testing (POCT) solutions in Table 1 (1–77), while addressing key issues and determining how the different solutions can help meet public health challenges. This article applies POC-geospatial concepts to disaster preparedness, emergency management, and public health challenges, such as outbreaks and their containment. The long-term mission is to prepare public health practitioners and laboratory medicine professionals to collaboratively employ geospatial tools and POCT in emergencies, epidemics, and disasters and to enable these specialists to make rapid diagnostic-therapeutic decisions at points of need.

**Table 1:** Geospatial science and point-of-care testing integrated solutions.

**Solution no.first authorJo./Yr./Ref**.	**Country, setting, or focus**	**Geospatial science topicTitle of paper(s) Geospatial synopsis and impact analysis**
**S1 [*****n*** **=** **12 designs]**		**Small-World Networks (SWNs) and regional topography**
**Kost** *Am J Clin Path* 2006 (1)	Phang Nga, Phuket, Krabi, and Trang Provinces in coastal Thailand; and vicinity of Louisiana State in the US	**Katrina, the Tsunami, and point-of-care testing: Optimizing rapid response diagnosis in disasters** We assessed how POCT can optimize diagnosis, triage, and patient monitoring during disasters. We surveyed 4 primary care units (PCUs) and 10 hospitals in provinces hit hardest by the tsunami in Thailand and 22 hospitals in Katrina-affected disaster areas. We compared how SWN properties in both countries could be used to improve POC and temporal responses to complex crises. We recommend handheld POCT, airborne critical care testing, and disaster-specific mobile medical units in SWNs worldwide in anticipation of future disasters and complex emergencies.
**Kost** *Point of Care* 2006 (2)	Public health vision	**Newdemics, public health, small-world networks, and point-of-care testing** Newdemics are defined as unexpected and disruptive problems that affect the health of large numbers of individuals in a crowded world. Newdemics demand dynamic value strategies in complex adaptive systems. Health professionals have to do more, and do it faster, but need also to practice evidence-based medicine. POCT allows demographic care units to continue serving critically ill clusters of people by relocating diagnostic, monitoring, and therapeutic resources through fast, patient-focused, and disease-specific evidence for decision making during outbreaks, complex emergencies, and disasters. Strategically modern POCT-SWNs will help optimize public health and newdemics outcomes in the 21st century world community of booming populations.
**Kost** *Point of Care* 2006 (3)	Mae Hong Son Province, Thailand	**The hill tribes of Thailand: Synergistic health care through point-of-care testing, small-world networks, and nodally flexible telemedicine** Hill tribes in Thailand approach 1 million people, about half Karens. The authors studied isolated Mae Hong Son Province in northwest Thailand near the Myanmar (Burma) border, where geodemographic research revealed overburdened health resources (938 people per hospital bed, 8721 per physician, 1068 per professional nurse, 4573 per technical nurse, 17,046 per pharmacist, and 5137 per primary care unit [PCU]). We investigated telemedicine, a nodally flexible option to improve SWN connectivity, which is planned to connect PCUs in the southern part of the province where seasonal rains make travel impractical. Geospatial analysis complements well the insertion of new POC technologies.
**Gundlapalli** *AMIA Symposium Proceedings* 2009 (4)	Salt Lake City, Utah, US	**Social network analyses of patient-healthcare worker interactions: Implications for disease transmission** Patients and healthcare workers (HCW)…represent a unique social network in which the risk of transmission of an infection is considered to be higher for both…. In sum, the patient-HCW network exhibits strong small world property….[that must be considered] to prevent the spread of infectious diseases in healthcare settings.
**Kost** *Point of Care* 2010 (5)	Rural Isaan, Thailand	**Emergency cardiac biomarkers and point-of-care testing: Optimizing acute coronary syndrome care using small-world networks in rural settings** Point-of-care cardiac biomarker testing is warranted in rural SWNs to guide early therapy and to educate young physicians in the management of acute coronary syndromes. Handheld and small benchtop instruments can be implemented effectively in SWNs not just for faster rescue, triage, transport, and treatment of critically ill patients with acute myocardial infarction, but also for improved medical and economic outcomes. The research in this pivotal paper led to implementation of POC cardiac biomarker testing throughout Thailand in over 500 sites with over 700 instruments.
**Yu** *Point of Care* 2010 (6)	Haiti	**Future connectivity for disaster and emergency point of care** The admirable humanitarian efforts of more than 4,000 organizations substantially impacted the lives of earthquake victims in Haiti. However, the lack of connectivity and SWN strategies, combined with communication failures, during early stages of the relief effort must be addressed for future disaster preparedness. Figures show the epicenter and surrounding areas of destruction from the earthquake, a proposed field area network for victim information connectivity, and the lab-in-a-backpack rescue concept.
**Kost** *Point of Care* 2011 (7)	Buriram Province, Thailand	**Point-of-need hemoglobin A1c for evidence-based diabetes care in rural small-world networks: Khumuang Community Hospital, Buriram, Thailand** Rapid on-site HbA1c testing of up to 150 diabetic PCU patients per day, quickly and efficiently identified those who were poorly controlled. Unexpectedly, elevated HbA1c changed primary care strategy, pulling together a rotating team of physicians, nurses, and a pharmacist who adjust therapy and accelerate checks for albuminuria to prevent advancing disease, dialysis, and adverse outcomes. This SWN motivates public health leadership to invest in POC HbA1c monitoring and enables appropriate evidence-based diagnostic screening using new POC technologies, software, and concepts. These technologies must be capable of high volume urgent testing that matches patient workflow.
**Kleczkowski** *J R Soc Interface* 2012 (8)	United Kingdom [theoretical study]	**Searching for the most cost-effective strategy for controlling epidemics spreading on regular and small-world networks** The authors present a combined epidemiological and economic model for control of diseases spreading on local and SWNs. Treatment is only desirable if the disease spreads on a SWN with sufficiently few long-range links; otherwise it is optimal to treat globally. The effectiveness of local (ring-vaccination or culling) and global control strategies is analyzed by comparing the net present values of the combined cost of preventive treatment and illness.
**Kost** *Point of Care* 2012 (9)	Fundamental theory and principles	**Theory, principles, and practice of optimizing point-of-care small-world networks** A healthcare SWN evolves naturally from social interactions and population dynamics. The physical SWN(p), when transformed into a virtual time domain network, SWN(t), anticipates dynamics of successful responses and rescues. SWN(t) reveals why POCT has high impact during complex emergencies and natural disasters—rapid test results optimize therapeutic turnaround time locally, while accelerating overburdened care paths globally. Especially in regions of heterogeneous population clusters where people in need may not have immediate access to tertiary care facilities, the POC SWN concept will be enhanced by determining provincial priorities based on demographic resource scoring, by use of GISs, and by linking individual SWNs in broader regional collaborations for optimal resilience.
**Kost** J of Demography (Chulalongkorn University, Bangkok)2012 (10)	Chiang Rai Province, Northern Thailand and border regions of Laos PDR	**Human immunodeficiency virus, population dynamics, and rapid strategies for medical diagnosis in the northern most province of Thailand—Chiang Rai** Innovative, effective, and efficient HIV POC tests and viral load monitoring should be extensively implemented in province hospitals, primary care units, HIV clinics, and the home with self-testing, in order to meet the standard of care, to improve case discovery, and to facilitate evidence-based decision-making regarding therapy and its follow-up. Health facilities should be available in border areas and at entry points in order to perform HIV POC screening of migrants, tourists, traders, and traffickers. SWN analysis in Chiang Rai helped identify Laotian women crossing the Mekong River and bringing HIV to Thailand. These advances will help reduce the spread of HIV/AIDS.
**Kost** *Point of Care* 2013 (11)	Vision statement	**The final frontier for point of care: performance, resilience, and culture** Global harmonization of POC performance will accelerate progress by improving the quality, usefulness, and impact of rapid decision-making. Worldwide outreach and culturally sensitive POC strategies in SWNs will enhance standards of care, including crisis standards of care during public health pandemics, complex emergencies, and natural disasters.
**Kost***Global Point of Care* 2015 (12)	Overview of theory and applications of SWNs	**Using small-world networks to optimize preparedness, response, and resilience**Resiliency through use of POCT in SWNs changes future landscape by bringing evidence-based decision-making directly to sites of need in healthcare systems, which should not be thought of as separate and distinct from SWNs serving groups of people and geographic regions. Connectivity of the physical (*p*), temporal (*t*), and virtual (*v*) SWN domains generates resilient healthcare, a key practice principle. The transformation, *SWN(p)* ⇒*SWN(t)* ⇒*SWN(v)*, and the parallel progressive enhancement of SWNs occurs through the portability, accessibility, timeliness, and scalability of crucial knowledge.
**S2 [*****n*** **=** **3]**		**Space-Time Transformation, Hubs, and Asset Mapping**
**Kost** *Global Point of Care* 2015 (12)[Geospatial analysis section of chapter 49.]	Nan Province, Thailand	**Space-time transformation and the benefits of hubs** The authors transformed the Kalasin SWN(p) to its *SWN(t)*. Emergency medical system staff intuitively optimize ambulance paths when transporting patients in the SWN(p). Transformation of the SWN(p) to *SWN(t)* reveals isolated nodes (Tha Khan Tho), key hubs (Somdet), and challenging routes for community hospital clusters that fall within prolonged time isopleths. Positioning POC strategically in nodes, clusters, and hubs can enhance standards of care by reducing risks through evidence-based triage, monitoring en route, and targeting definitive treatment more quickly.The authors studied the impact of a strategic hub on the *SWN(t)* of Nan Province. The *SWN(t)* for the entire province shows transport times in minutes, some quite prolonged. In contrast, a cluster of five community hospitals can send patients to the regional hub at Pua Crowne Prince Hospital in a tiered system of referral. Shorter transport times to the hub combined with rapid POCT yield self-sufficiency and support specialists who address acute medical and surgical problems by delivering timely treatment.
**Girdwood** *PLoS ONE* 2019 (13)	Zambia sample transportation network	**Optimizing viral load testing access for the last mile: Geospatial cost model for point of care instrument placement** The authors used a combination of both on-site POCT and placement at facilities acting as POC hubs. A location allocation model was used to identify POC hubs. An optimal combination of both on-site placement and the use of POC hubs can reduce the cost per test by 6–35% by reducing transport costs and increasing instrument utilization. Please see the entry below under “S4. GIS-POCT Field Research” for additional details.
**MacKenzie** *Front Publ Hlth* 2019 (14)	Public health, US	**A public health service-learning capstone: Ideal for students, academia and community** Under public health capstone competencies and assignments, the authors recommend mapping community healthcare, assessing community resources, and synthesizing community strengths and gaps. They define the task as a windshield survey of the geographic area in which agency is situated to include environmental factors that influence the lives of the population served. Then, by use of community asset maps, they develop education service proposals and recommend population-level approaches to address public health problems.
**S3 [*****n*** **=** **7]**		**Spatial and geospatial care pathsTM (SCPs)**
**Kost** *e-Journal IFCC* 2014 (15)	Limited-resource and other healthcare settings	**Principles of point of care culture, the spatial care path**™**, and enabling community and global resilience** In contrast to the past where attention has been placed on emergency departments, hospitals, and referral centers, the SCP starts with the patient and guides him or her through an efficient strategy of care in SWNs defined by local geography and topology, long-standing customs and cultural norms, public health jurisdictions and professional behavior, and geographic information systems. The SCP facilitates an essential balance of prevention and intervention in public health and shifts future focus to the patient, empowerment, and primary care within the context of POC culture.
**Kost** *Amer J Dis Med* 2015 (16)	Strategic planning for epidemics	**The Ebola Spatial Care Path**™**: Accelerating point-of-care diagnosis, decision making, and community resilience in outbreaks** POCT is facilitating global health. Now, global health problems are elevating POCT to new levels of importance for accelerating diagnosis and evidence-based decision making during disease outbreaks. The authors present a vision where POCT accelerates an Ebola SCP and future molecular diagnostics enable facilitated-access self-testing; design an alternate care facility for the SCP; innovate an Ebola diagnostic center; and propel rapid POCT to the frontline to create resilience that stops future outbreaks.
**Kost** *Point of Care* 2016 (17)	Reenergizing vision	**Spatial Care Paths**™ **strengthen links in the chain of global resilience: Disaster caches, prediabetes, Ebola virus disease, and the future of point of care** By identifying weak links in the chain of community resilience, SCPs upscale key unfulfilled needs, discover new ideas for innovation-invention, bolster educational outreach, and improve patient access to evidence-based primary, emergency, and hospital care. Strong collaborative initiatives can foster activism in the global community. It is time for insightful leadership and participative outreach to bridge professional disciplines, span different countries, and steward POC into a brilliant new future.
**Kost** *Point of Care* 2017 (18)	Underserved populations	**Diabetes Spatial Care Paths**™**, leading edge HbA1c testing, facilitation thresholds, proactive-preemptive strategic intelligence, and unmanned aerial vehicles in limited-resource countries** By taking advantage of strategic intelligence, in the form of a SCP for diabetes in limited-resource countries, and moving to primary care, the flow of knowledge emanating directly from patients will help public health nurses, primary care staff, and multidisciplinary physicians, some working via telemedicine, to proactively and preemptively reduce diabetes complications by means of evidence-based, cost-effective decision making closer to patient homes. Innovative monitoring and treatment will fulfill expectations for high-quality efficient personalized care, even self-monitoring essential to the management of a chronic condition, thus transforming standards of care to appropriately embrace and empower POC culture.
**Kost** *Point of Care* 2018 (19)	Hue Province, Central Vietnam	**Point-of-care diagnosis of acute myocardial infarction in Central Vietnam: International exchange, needs assessment, and Spatial Care Paths** ™Central Vietnam must improve rapid diagnosis and treatment of AMI patients. Early upstream POC cardiac troponin testing on SCPs will expedite transfers directly to hospitals capable of intervening, improving outcomes following coronary occlusion. Point-of-care coordinator certification and financial support will enhance standards of care cost-effectively. Training young physicians pivots on high-value evidence-based learning when POC cardiac troponin T/troponin I biomarkers are in place for rapid decision making, especially in emergency rooms.
**Ventura** *Point of Care* 2019 (20)	Hue Province, Central Vietnam	**Rapid diagnosis and effective monitoring of diabetes mellitus in Central Vietnam: point-of-care needs, improved patient access, and spatial care paths for enhanced public health** The lack of HbA1c testing in Central Vietnam decreases the ability to monitor patient response to treatment in limited-resource settings. During patient-provider encounters, POC HbA1c may be used to achieve more timely treatment changes to improve patient outcomes. When placed in low resource rural settings where physicians face high volume workloads, rapid onsite HbA1c testing can quickly and effectively identify patient glucose control or lack thereof. Accessible online training, public health teamwork, an appropriately detailed spatial care path (presented in the paper), and POC measurement of HbA1c with target levels set for the Vietnamese population, have high probability of strategically and dynamically balancing needs fulfillment and scarce resources in Central Vietnam.
**Kost** *Tri∙Con Symposium* 2019 (21)	Hualien County, Taiwan; Palawan, the Philippines; Isaan, Thailand; and Central Vietnam	**Point-of-care cardiac biomarkers in Vietnam, the Philippines, Taiwan, and Thailand** The speaker compared and contrasted different geographic settings and demonstrated how GIS analysis could position POC cardiac biomarkers to eliminate delays in diagnosis, improve patient access, accelerate response time, and enhance cardiac care, especially in coastal Hualien County, Eastern Taiwan, and remote Palawan Island, the Philippines, both highly linear topographies; and in Isaan, Thailand, and Central Vietnam, both extremely limited-resource. In these settings, new *geospatial care paths^*TM*^*, which take into account regional demography, can be fashioned to encompass features of population clusters, migration fluxes, local POCT service hubs, and potential coastal displacements from rising ocean levels associated with global warming.
**S4 [*****n*** **=** **13]**		**Geographic information systems (GIS)** **+** **point-of-care testing**
**Grusky** *Behav Med* 2010 (22)	Los Angeles, California, US	**Staff strategies for improving HIV detection using mobile HIV rapid testing** The authors created maps using geographic GIS data on 93 mobile testing unit (MTU) locations and 2,003 AIDS cases. MTU testing locations were clustered near high AIDS rate areas. Staff strategies that were used included keeping clients with them while rapid test results were being processed and adjusting to clients' schedules when arranging for picking up test results. GIS findings and client risk data support the CDC policy of implementing MTUs and rapid testing in large urban communities with high AIDS rates.
**Goswami** *BMC Infect Dis* 2011 (23)	Wake County, North Carolina, US	**Feasibility and willingness-to-pay for integrated community-based tuberculosis testing** Integrated testing for TB, HIV, and syphilis was performed in neighborhoods identified using GIS-based disease mapping. TB testing included skin testing and interferon gamma release assays. Successful integrated testing programs in high risk populations will likely require one-visit diagnostic testing and incentives.
**Ferguson** *Point of Care* 2012 (24)	Fundamental hypothesis	**Geographic information systems can enhance crisis standards of care during complex emergencies and disasters: A strategy for global positioning system-tracked, H**_**2**_ **fuel cell-powered, and knowledge-optimized point-of-care medical intelligence** The authors hypothesize that a medical GIS can improve medical response during complex emergencies and disasters by facilitating the strategic placement and management of POC technologies within a SWN. The GIS-POC-SWN approach will speed informed decision making, optimize POC medical intelligence, and enhance crisis standards of care.
**Nagata** *Prehosp Dis Med* 2012 (25)	Fukushima, Japan	**Use of a geographic information system (GIS) in the medical response to the Fukushima nuclear disaster in Japan** The Great East Japan Earthquake occurred on March 11, 2011. In the first 10 days after the event, information about radiation risks from the Fukushima Daiichi nuclear plant was unavailable, and the disaster response, including deployment of disaster teams, was delayed. Beginning on March 17, 2011, the Japan Medical Association used a geographic information system (GIS) to visualize the risk of radiation exposure in Fukushima. This information facilitated the decision to deploy disaster medical response teams with POCT instruments on March 18, 2011.
**Alegana** *Spatial Spatio-temp Epi* 2013 (26)	Northern Namibia	**Estimation of malaria incidence in northern Namibia in 2009 using Bayesian conditional-autoregressive spatial–temporal models** A spatial-temporal model was used to identify constituencies with high malaria incidence to guide malaria control. Rapid diagnostic tests were used to examine blood samples from most patients at primary health facilities although a few, mostly at tertiary facilities, were examined using microscopy. The spatial distribution of reported cases, including suspected cases adjusted for test positivity rates, indicates higher caseloads in the northern regions.
**Yao** *Health Place* 2014 (27)	Rural Mozambique	**Spatial and social inequities in HIV testing utilization in the context of rapid scale-up of HIV/AIDS services in rural Mozambique** Applying GIS-based methods and multilevel regression analysis to unique longitudinal three-wave survey data from rural Mozambique, the authors investigated the impact of a rapid expansion of HIV-related services on access to and utilization of HIV testing. The results illustrate the declining importance of spatial barriers to utilization of HIV testing services as these services expanded. In addition, the expansion of HIV-related services decreased the spatial variability of HIV testing among the survey respondents.
**Ferguson** *Global Point of Care* 2015 (28)	Summary of principles of GISs when integrated with POCT	**Use of geographic information systems for placement and management of point-of-care technologies in small-world networks** Point-of-care technologies afford first responders with the mobility to deliver diagnostic testing at the site of care, and because they do not rely on conventional infrastructure, are more robust for use in disasters and complex emergencies. Healthcare systems are built on geographic relationships between patients and resources that reliably provide care to them and can be thought of as SWNs. A GIS allows us to view and analyze spatial relationships among entities to draw conclusions. A GIS can quantify SWNs leading to informed decisions on improving the healthcare systems in the context of day-to-day and disaster medical management.
**Ferguson** *A Spatial Model Report* 2015 (29)	Comparison of GIS applications in five nations	**Streamlining health access through point of care technologies: a spatial model** Rapid and accurate diagnoses drive evidence-based care in health systems. Using GISs we can understand how populations utilize health networks, visualize their inefficiencies, and model alternatives…and also help evaluate alternative POC diagnostic placement strategies compared to current health access. We present visual logistics from GIS analyses in the Eyre Peninsula, Australia; Pernambuco State, Brazil; Palawan Island, Philippines; Hualien County, Taiwan; and Nan Province, Thailand. Importantly, use of POC cardiac biomarkers (cTn T and I) in Brazil will provide more equitable care, and use of POC HbA1c in south Australia will improve access to care and monitoring of therapy for Indigenous Aboriginal populations with high prevalence of serious diabetes, and in the case of homes more than 1 h transit from South Australian and Aboriginal Health Facilities, will fit culturally to encourage personalized medicine, improved outcomes, and less dialysis.
**Ferguson** *Int J Hlth Geogr* 2016 (30)	Rural Isaan, Thailand	**Using a geographic information system to enhance patient access to point-of-care diagnostics in a limited-resource setting** Geospatial analyses derive high impact by improving alternative diagnostic placement strategies in limited-resource settings and by revealing deficiencies in health care access pathways. GIS provides a platform for comparing relative costs, assessing benefits, and improving outcomes. This approach can be implemented effectively by health ministries seeking to enhance cardiac care despite limited resources.
**Larroca** *Malaria J* 2016 (31)	Districts with highest prevalence of malaria, Uganda	**Malaria diagnosis and mapping with m-Health and geographic information systems (GIS): evidence from Uganda** Affordable remote malaria diagnosis and mobile health can help to decongest health facilities, reducing costs and contagion. The authors discuss rapid diagnostic tests, their limitations, advantages, and impact in conjunction with m-Health. Mapping by means of GIS analysis could provide real-time and geo-localized data transmission, improving anti-malarial strategies in Uganda.
**Lin** *Point of Care* 2017 (32)	Hualien County, East Coast, Taiwan	**Bio-innovation in Taiwan, the first survey of point-of-care professional needs, and geospatially enhanced resilience in at-risk settings** The authors analyzed distance/ time/economic metrics for POC diagnosis in Hualien, an eastern seaboard county vulnerable to typhoons. Geospatial analysis showed that POCT can speed acute response in rural areas of Hualien County. Priorities include rural areas and vulnerable populations.
**Girdwood** *PLoS ONE* 2019 (13)	Zambia sample transportation network	**Geospatial cost model for point of care instrument placement** Viral load (VL) monitoring programs are now facing the challenge of providing access to remote facilities. For the hardest-to-reach facilities in Zambia, the authors compared the cost of placing POC VL instruments at or near facilities to the cost of an expanded sample transportation network to deliver samples to centralized laboratories. ArcGIS 10.5 (ESRI) was used to run different algorithms to identify candidate POC facilities, select facilities for POC placement, and model the different scenarios. POC VL testing reduces costs of expanding access to the hardest-to-reach populations, despite the cost of equipment and low patient volumes. An optimal combination of both on-site placement and the use of POC hubs can reduce the cost per test by 6–35% by reducing transport costs and increasing instrument utilization.
**Kuupiel** *BMC Public Health* 2019 (33)	Ghana	**Geographic accessibility to public health facilities providing tuberculosis testing services at point-of-care in the upper east region, Ghana** There is poor geographic accessibility to public health facilities providing TB testing services at the POC in the upper east region of Ghana. The authors assembled detailed spatial data on all 10 health facilities providing TB testing services at the POC, and landscape features influencing journeys. These data were used in a geospatial model to estimate actual distance and travel time from the residential areas to health facilities providing TB testing services. Maps displaying the distance values were produced using ArcGIS Desktop v10.4. Targeted improvement of rural public health clinics in the upper east region and TB testing services at the POC are highly recommended.
**S5 [*****n*** **=** **7]**		**Isolation Laboratories, Diagnostics Isolators, and Mobile Laboratories for Highly Infectious Diseases**
**Hill** *Lab Med* 2014 (34)	Emory University, Atlanta, GA	**Laboratory test support for Ebola patients within a high-containment facility** The authors present an isolation laboratory designed collaboratively with the CDC several years prior to receiving two Ebola patients and list POC tests used inside. To avoid aerosol exposure, no centrifugation was performed. Prothrombin time (PT) testing to document coagulation status was used “off label,” that is, not FDA cleared for Ebola patients. The experience highlights the need for (a) FDA-cleared tests, (b) compact instruments, (c) direct whole-blood measurement, (d) consolidation of test clusters appropriate for the support of patients critically ill with highly infectious diseases, and (e) spatially discrete “safe houses” for POCT.
**Kost** *Amer J Dis Med* 2015 (16) *Clin Lab Intl* 2015 (35) *Expert Rev Mol Diagnostics* 2015 (36)	Southeast Asia (Bangkok, Thailand) and other settings at risk worldwide	**The Ebola Spatial Care Path**™**: Accelerating point-of-care diagnosis, decision making, and community resilience in outbreaks** (16) **The Ebola Spatial Care Path**™**: Point-of-care lessons learned for stopping outbreaks** (35) **Molecular detection and point-of-care testing in Ebola virus disease and other threats: a new global public health framework to stop outbreaks** (36) The authors designed and built several isolation laboratories for highly infectious diseases in hospitals in anticipation of Ebola outbreaks hitting Southeast Asia. POCT instruments are operated inside a biosafety cabinet within the controlled airflow isolation area by personnel wearing PPE, which is donned in a changing area within the isolation laboratory. POC tests include critical care test clusters. Personnel doff PPE in a separate area under strict precautions that avoid contamination through autoclaving. Specimens are passed into the isolation laboratory through a double door isolator. The essence of the approach is discrete spatial isolation and simultaneous control of environmental conditions. Thus, the isolation laboratory enhances safety, and temperature and humidity controlled to within reagent and instrument specifications to simultaneously assure accurate POC test results.
**Shorten** *PLoS Negl Trop Dis* 2016 (37)	West Africa; London, England	**Diagnostics in Ebola virus disease in resource-rich and resource-limited settings** Figures present clever isolator designs with POCT inside used in Sierra Leone and detail POC instruments. The authors conclude that limited access…contributed to the initial failure to contain the outbreak in West Africa.…future outbreaks will be…terminated more efficiently…through greater access to portable, easy-to-use diagnostic assays.
**Boonlert** *Point of Care* 2006 (38)	Ten provinces in Northern Thailand	**Point-of-care testing on a mobile medical unit in northern Thailand: Screening for hyperglycemia, hyperlipidemia, and thalassemia trait** The Mobile Medical Unit of Naresuan Faculty and University Research and Innovation facilitates health care delivery for people in rural areas of Thailand by transporting a health care team and small laboratory directly to where they live and can be used efficiently with POCT for hyperglycemia, hyperlipidemia, and thalassemia trait screening in public health. POCT also includes tests for WBC, and infectious diseases, such as HIV and hepatitis B virus, and could be outfitted with a diagnostics isolator for the rapid diagnosis of highly infectious diseases.
**Diers** *Med Sante Trop* 2015 (39)	Mali, West Africa	**Mobile laboratories for rapid deployment and their contribution to the containment of emerging diseases in Sub-Saharan Africa, illustrated by the example of Ebola virus disease** The authors propose a framework in which these mobile laboratory units can strengthen epidemiological surveillance and contribute to containing outbreaks of emerging diseases in sub-Saharan Africa. Rapidly deployable laboratory units can bring the diagnosis closer to the outbreak site and significantly shorten the time to delivery of results, thus facilitating epidemic containment.
**Mansuy** *Lancet Infect Dis* 2015 (40) **De la Vega** *ERAIT* 2016 (41)	West Africa	**Mobile laboratories for Ebola and other pathogens** (40) **Diagnosis and management of Ebola samples in the laboratory** (41) The authors present outbreak response workflow from the point of view of mobile laboratories during the West African Ebola outbreak of 2014–2016. Mobile laboratories located in areas where Ebola was spreading in west Africa drastically reduced the time between collection of biological specimens and return of results, making them much more effective than central laboratories located far from the patients. The shorter the delay in obtaining a test result, the better confirmed cases can be managed, and cases of potential but unconfirmed disease can be monitored, reducing virus transmission. Additionally, rapid virological testing of biological samples from the deceased helps manage secure burials. A reactive network of mobile laboratories should offer differential diagnoses for Ebola, malaria, shigellosis, cholera, and typhoid in context of local epidemiological data.
**Racine** *Human Vaccin Immunother* 2019 (42)	Canada	**Challenges and perspectives on the use of mobile laboratories during outbreaks and their use for vaccine evaluation** Mobile laboratories provide diagnostic capabilities for routine surveillance and patient identification during an outbreak and should be used in the evaluation of novel vaccines and therapeutics in remote locations. Clinical mobile laboratories include similar diagnostic capabilities as outbreak response mobile labs, but also include additional POC instruments. Failure to deploy a clinical mobile laboratory when administering a novel biological product in a remote location limits any collected scientific data and could ultimately undermine clinical development and availability of life-saving interventions.
**S6 [*****n*** **=** **1]**		**Alternate Care Facilities (ACFs)**
**Kost** *Am J Dis Med* 2011 (43) *Am J Dis Med* 2015 (16) *A Practical Guide to Global POCT* 2016 (44)	Fundamental designs and isolation laboratories built into hospitals in Bangkok, Thailand	**Enhancing standards of care using innovative point-of-care testing** (43) **The Ebola Spatial Care Path**™**: Accelerating point-of-care diagnosis, decision making, and community resilience in outbreaks** (16) **Point-of-care testing for Ebola and other highly infectious diseases: Principles, practice, and strategies for stopping outbreaks** (44) The authors designed an ACF to integrate SCP principles for urgent Ebola care. The floor plan embeds POCT to be used in support of patients being screened for EVD and for those seriously ill and in need of critical care while in isolation. The ACF is free-standing, modular, expandable, and independent of hospital facilities to avoid contagion as an integrated community resource that increases efficiency and decreases risk, while using POCT to accelerate diagnosis and decision making. PPE-trained staff oversee diagnostic instruments. Modular partitions can be moved to increase the number of individual isolation rooms for suspected, but not confirmed, patients. The overall gross dimensions can be enlarged to increase capacity. ACFs can be replicated to meet triage needs anywhere for quarantine of patients suspected of having highly infectious diseases during outbreaks and epidemics. Geospatial examples are provided showing the ACF in Indonesia and the logic of use for cyber-point of care responses to a complex emergency, disaster, or public health crisis.
**S7 [*****n*** **=** **11]**		**Roaming POC—Airborne, Ambulances, Space, and Wearables**
**Herr** *Amer J Clin Path* 1995 (45)	Helicopters	**Airborne and rescue point-of-care testing** The i-STAT Portable Clinical Analyzer was used on 81 patients transported by the flight crew. The tests performed in the helicopter included sodium, potassium, glucose, and hematocrit/hemoglobin concentrations. Fifteen (18.5%) of the patients were treated with transfusions, glucose, or insulin based on the Portable Clinical Analyzer results. Other identified needs include blood gas analysis and use of POCT in the fixed-wing environment.
**Davey** *Air Med J* 2001 (46)	Pediatric air transport	**Changes in pCO**_**2**_ **during air medical transport of children with closed head injuries** Mechanical ventilation appears mandatory, and monitoring CO_2_ in transit (end-tidal or preferably POCT) should further reduce the likelihood of secondary complications from cerebral ischemia.
**Di Serio** *Clin Chem Lab Med* 2010 (47)	Air ambulances	**Laboratory testing during critical care transport: point-of-care testing in air ambulances** Real-time results during transport of critically ill patients must be considered to be an integral part of the patient care process and excellent channels of communication are needed between the intensive care units, emergency medical services and laboratories.
**Louie** *Am J Dis Med* 2013 (48)	Flight from Hawaii to the Marshall Islands and transport of patient back to Hawaii	**Effects of environmental conditions on point-of-care cardiac biomarker test performance during a simulated rescue: implications for emergency and disaster response** In a simulated rescue of Marshal Islands patients with chest pain flown to Hawaii for intervention, short-term temperature elevation produced falsely lower cTnI results. Some stressed cTnI measurements falsely reported normal levels when control results indicated alert values potentially leading false-negative diagnosis of an acute myocardial infarction.
**Tideman** *Med J Aust* 2014 (49)	Rural South Australia	**Impact of a regionalized clinical cardiac support network on mortality among rural patients with myocardial infarction** An integrated cardiac support network incorporating standardized risk stratification, POC troponin testing, and cardiologist-supported decision making was implemented in non-metropolitan South Australia using standardized risk stratification and evidence- based treatment protocols; POC whole-blood troponin T; an on-call consultant cardiologist with redundancy to ensure response within 10 min with facsimile-based electrocardiogram interpretation; and facilitation of transfer to metropolitan hospitals by the Royal Flying Doctor Service with emergency medical retrieval team support if deemed necessary. The authors observed improvement in 30-day mortality for patients presenting to rural hospitals and diagnosed with myocardial infarction. These interventions closed the gap in mortality between rural and metropolitan patients in South Australia.
**Sorensen** *Global Point of Care* 2015 (50) **Rasmussen** *Eur Heart J Acute Cardiovasc Care* 2017 (51)	Netherlands ambulances	**Prehospital application of cardiac biomarkers for decision support of patients with suspected acute myocardial infarction** (40) **Predictive value of routine point-of-care cardiac troponin T measurement for prehospital diagnosis and risk-stratification in patients with suspected acute myocardial infarction** (41) Prehospital diagnosis of patients with acute coronary syndromes enables referral to optimal treatment in a timely manner. In ST-elevation myocardial infarction several studies documented prehospital ECG reduces time to treatment, thereby reducing mortality and morbidity. Increasing evidence from qualitative and quantitative POC cardiac troponins in ambulances provides important triage and prognostic information. In the most recent paper, patients with suspected acute myocardial infarction and a prehospital POC cardiac troponin T ≥ 50 ng/l have poor prognosis irrespective of the final diagnosis, thus high-risk even before hospital arrival, allowing re-routing directly for advanced care at an invasive center.
**NIH-NASA Inaugural Panel** 2010 and 2011	International Space Station—seminal POC experiments	**International Space Station (Biomed-ISS), NASA-NIBIB 1st Collaboration (2010)** The first Joint NIH and NASA Panel (2010) and Special Emphasis Panel, ZEB1 OSR-E M1 S (2011), NIBIB, NIH, reviewed collaboratively experiments and POC technologies designed for microgravity in the International Space Station [GK panelist].
**Kost** PCQACL 12th Annual Conv., Manila, Philip.2015 (52)	Mars Colony	**How POCT Improves Care and Educates Physicians: Exciting Contemporary Examples and Innovative Opportunities, including Point-of-Careology on Mars** The speaker identified needs for diagnostic testing to support individuals inhabiting the Mars colony, identified potential ways POCT could be positioned within the compound, and suggested appropriate test clusters that should be available on Mars.
**Canadian Consortium**2017 (53)	International Space Station—wearable sensors and POCT	**Bio-Analyzer and Bio-Monitor: Near real-time biomedical results from space to Earth** According to the Canadian Space Agency, the Bio-Monitor enables “smartshirt” wearable sensors for physiological monitoring (ECG, BP, RR, skin temperature, O_2_ saturation, and physical activity), while the Bio-Analyzer provides test results from blood, urine, and saliva samples from space within 2–3 h reducing need to freeze and return samples to Earth. It facilitates blood draws by using a finger prick sample, eliminates need for a standard needle; maintains quality of the sample; obviates need for frozen samples; enables new testing, such as specific cell counts; and frees up valuable storage space on board the International Space Station and on cargo ships that transport frozen materials back to Earth. [See http://asc-csa.gc.ca/eng/sciences/bio-monitor.asp and http://asc-csa.gc.ca/eng/missions/expedition58/about-the-mission/mission-highlights.asp]
**Roda** *Biosens Bioelectronics* 2018 (54)	International Space Station—portable and wearable biosensors	**Chemiluminescence-based biosensor for monitoring astronauts' health status during space missions: Results from the International Space Station** (54) **Advanced biosensors for monitoring astronauts' health during long-duration space missions** (55)
**Zangheri** *Biosens Bioelecrtronics* 2019 (55)		The authors report on the state of the art of diagnostic instrumentation, including portable and wearable biosensors, for monitoring astronaut health during long-duration space missions,. There is strong demand for simple analytical devices that astronauts can use to perform clinical chemistry analyses. A biosensor used successfully by an Italian astronaut during the VITA mission, July-December 2017, demonstrated the feasibility of performing sensitive lateral flow immunoassay analysis of salivary cortisol down to 0.4 ng/mL directly onboard the International Space Station.
**S8 [*****n*** **=** **4]**		**Connected and Wireless POCT Outside Hospitals**
**Kamanga** *Malaria J* 2010 (56)	Rural health centers, Zambia	**Rural health centers, communities and malaria case detection in Zambia using mobile telephones: a means to detect potential reservoirs of infection in unstable transmission conditions** Adequate supplies of rapid diagnostic tests are essential in health centers. Mobile telephones facilitate case detections in multiple locations, thereby saving time. The system can be expanded throughout the country to support rapid strategic targeting of interventions.
**Laksananasopin** *Global Point of Care* 2015 (57)	Muhima Hospital, Kigali, Rwanda, and mobile sites	**Integrating diagnostics tests and connectivity to enable disease diagnosis and tracking in remote settings** Mobile devices for detecting disease markers in patients enhance health around the world, as well as empowering field workers in times of emergencies, disasters, and public health crisis, such as disease outbreaks. The authors invented a low-cost technology with miniaturization for performing all essential functions of enzyme-linked immunosorbent assays with cell phone and satellite connectivity. Results are synchronized in real time with electronic health records via a Global System for Mobile Communications satellite, in order to bring better healthcare to resource-poor and decentralized settings.
**Connect Diagnostics Co., Ltd**.Dir. Mgr.: Arirat Banpavachit [ariratb@connectdiagnostics.com]2019	Bangkok, Thailand, and surrounds	**“LINK” brand in 3 models, LINK LIS, LINK POC, and LINK INT, provides out-of-hospital POCT connectivity systems for POCT** Unique software systems fill the connectivity gap between the patient's home or primary care by linking these locations with the sites of professional oversight to create comprehensive patient management using self-testing and other POC modalities in the spatial domain of comprehensive community care.[Access at https://connecthealthshop.com/]
**Smith** *Lab Chip* 2019 (58)	Resource-limited settings, Southern Africa	**Wireless colorimetric readout to enable resource-limited point-of-care** The authors present a scalable, generic wireless color detector for POC diagnostics in resource-limited settings. The challenges faced in these settings have limited the effectiveness of POC diagnostics. By combining paper-based diagnostics and printed electronics with Southern African clinic perspectives, a mass-producible, low-cost, paper-based solution for result readout and wireless communication was developed.
**S9 [*****n*** **=** **3]**		**Unmanned Aerial Vehicles (UAV, Drones)**
**Amukele** *J Clin Microbiol* 2016 (59) *PLoS ONE* 2017 (60) *Am J Clin Pathol* 2017 (61) *J Appl Lab Med* 2019 (62)	Mock-up flights in the vicinity of Johns Hopkins University, Baltimore, MD, US	**Drone transport of microbes in blood and sputum laboratory specimens** (59) **Can unmanned aerial systems (drones) be used for the routine transport of chemistry, hematology, and coagulation laboratory specimens?** (60) **Drone transport of chemistry and hematology samples over long distances** (61) **Current state of drones ion healthcare: Challenges and Opportunities** (62) Transportation of laboratory specimens does not affect the accuracy of routine chemistry, hematology, and coagulation tests results, except slightly poorer precision. Changes in glucose and potassium were consistent with the magnitude and duration of the temperature difference between flown and stationary samples. Long drone flights are feasible but require stringent environmental controls. Times to recovery, colony counts, morphologies, and matrix-assisted laser desorption ionization-time of flight mass spectrometry-based identifications were similar for all microbes.
**Priye** *Anal Chem* 2016 (63)	College Station, TX, US	**Lab on a drone: Toward pinpoint deployment of smartphone-enabled nucleic acid-based diagnostic for mobile healthcare** The authors introduce portable biochemical analysis for rapid field deployment of nucleic acid-based diagnostics using quadcopter drones, isothermally performing PCR with a single heater, enabling 5 V USB sources. Time-resolved fluorescence detection and quantification uses a smartphone camera and integrated image analysis app. Sample preparation leverages the drone's motors as centrifuges via 3D printed snap-on attachments. The DNA/RNA system costs ~$50, enabling deployment to field sites. Successful in-flight replication of Staphylococcus aureus and λ-phage DNA targets is <20 min. Rapid in-flight assays with smartphone connectivity eliminates delays between sample collection and analysis.
**Kost** *Pont of Care* 2017 (18)	Mae Hon Son Province by the Myanmar border, Thailand	**Diabetes Spatial Care Paths**™**, leading edge HbA1c testing, facilitation thresholds, proactive-preemptive strategic intelligence, and unmanned aerial vehicles in limited-resource countries** Please see Kost et al. (17) under Solution 3 for a plan of drone use to transport specimens from remote border and isolated areas to diagnostic centers. Note that for drones that do not land at the target, but instead parachute payloads to the ground, transporting specimens from remote sites to referral centers requires launch capability in the remote sites.
**S10 [*****n*** **=** **7]**		**Geospatial Practice—Demographic Care Unit Resource Scoring, Geographic Risk Assessment, National POCT Policy and Guidelines, and Point-of-Careology**
**10.A**.		**Demographic Care Unit Resource Scoring**
**Kost** *The Demographic Dividend* 2005 (64) **Kost** *J Demography* (Chulalongkorn University, Bangkok)2011 (65)	Thailand Provinces	**Minimizing health problems to optimize the demographic dividend: The role of point-of-care testing** (64) **A new demographic strategy for point-of-need medical testing: Linking health resource scores, poverty levels, and care paths** (65) The authors invented a scoring metric comprising health resources, poverty levels, and diagnosticians to alleviate inequities in provinces with overburdened health systems and/or critical poverty. Scores reflect lack of primary care units, hospital beds, medical doctors, registered nurses, technical nurses, pharmacists, and medical technologists. The higher the score, the worse the situation. DCU clusters with high scores for health resources, high ratios of poor people, high numbers of poor people, and deficiencies in medical technologists were located mainly in the northeast, where POCT and unique new care paths can have a significant impact on improving outcomes and cost-effectiveness. Geodemographic linking of POCT and needs reveals where to implement fast mobile medical diagnostics and care paths that improve standards of care.
**10.B**.		**Geographic Risk Assessment**
**Kost** *Point of Care* 2012 (66)	Bangkok, Thailand	**Diagnostic testing strategies for health care delivery during the Great Bangkok Flood and other weather disasters** Feasibility of POCT was demonstrated in previous flood episodes (e.g., Hurricane Katrina) and again during the Great Bangkok Flood, although on a limited basis. Preparation, training, mobility, and deployment were challenges. In addition, some medical problems required sophisticated analytical methods, such as the diagnosis of Leptospirosis by PCR, not yet amenable to testing directly at the site of need. Unmet needs ensure a bright future for innovators who develop new POC solutions and increase the mobility of diagnostic services for weather disasters. Global warming will bring more floods. Risk assessment will mitigate damage in terms of both human and economic losses.
**Kost** *Point of Care* 2012 (67)	Phang Nga Province, South Thailand	**Strategic point-of-care requirements of hospitals and public health for preparedness in regions at risk** The authors studied health resources and POCT requirements for urgent, emergency, and disaster care in Phang Nga Province, Thailand, after the tragic 2004 Andaman Sea Tsunami; determined instrument design specifications through a direct needs assessment survey; described POC test menus useful in the SWN; and assessed strategies for preparedness. Respondents selected complete blood cell count, electrolytes/ chemistry, blood type, oxygen saturation (by pulse oximeter), hematocrit, and microbiology as top priorities and preferred direct blood sampling with cassettes. Cardiac biomarkers were important in alternate care facilities. *Staphylococcus aureus*, SARS, *Streptococcus pneumoniae*, and hepatitis B virus were top infectious disease problems. Temperature, vibration, humidity, and impact shock were four important environmental factors during extreme conditions. These data tell us how to integrate POCT in disaster situations.
**Kost** *Point of Care* 2013 (68)	Phang Nga Province, South Thailand	**Point-of-care testing value proposition for disaster preparedness in small-world networks: Post-Tsunami Phang Nga Province, Coastal Thailand** This study evolved a systematic approach to risk assessment and identified geographic sites at high risk in the event of a new Tsunami. The 2004 earthquake/Tsunami devastated Southeast Asia. The authors studied POCT and O_2_ saturation monitoring in Phang Nga, the hardest hit southern coastal province, to develop preparedness strategies for low-resource SWNs. Early 2005, they surveyed 4 provinces, then, in 2007–2011, focused on Phang Nga with new field/phone/mail/e-mail/fax surveys of 7 primary care units, all 7 community hospitals, and both regional hospitals. They used short- and long-form Thai surveys, photo documented instruments, and assessed resources. Overall, preparedness for medical testing improved significantly. Value proposition strategies built on post-Tsunami advances enhance SWN POCT preparedness, as well as daily emergency care. Daily use of POCT improves chances of high quality response during crises, if the POCT is positioned shrewdly in vulnerable geographic sites.
**Oxford University**2014 (69, 70)	Sub-Saharan Africa	**Sub-Saharan sites of high risk for Ebola virus disease contagion** Investigators mapped the risk of Ebola infection in Sub-Saharan Africa from the west coast (Guinea, Liberia, Sierra Leon) across the DRC to the east coast (Somalia, Kenya, Tanzania). The risk map (below) helps design spatial grids comprising SWNs, GISs, and topomaps with POCT embedded at essential nodal points to help contain Ebola outbreaks. 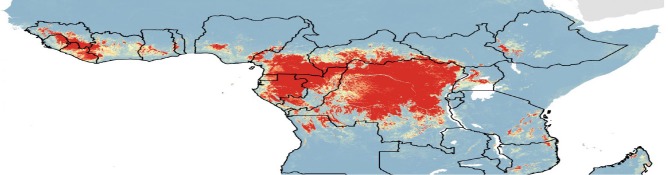 [Access the Ebola risk map at http://www.ox.ac.uk/news/2014-09-08-risk-ebola-emergence-mapped]
**10.C**.		**National POCT Policy and Guidelines**
**Baizurah** *Ministry of Public Health Document* 2012 (71)	Malaysia	**National Point of Care Testing Policy and Guidelines** Malaysian consensus committee produced a unique geopolicy directive that encompasses two widely separated geographic areas, the mainland and the states of Sabah and Sarawakon on the island of Borneo, to harmonize POCT policy and guidelines under one national roof. The document was prepared under the helm of Dr. Baizurah by a national consensus committee and published in English (only) by the Malaysian Ministry of Public Health.
**Kost** *Global Point of Care* 2015 (72)	Worldwide	**National point of care testing policy and guidelines in Malaysia, standards of care, and impact worldwide** Comprehensive background and interpretive analysis co-authored with Dr. Baizurah in conjunction with the National Point of Care Testing Policy and Guidelines document above.
**S11 [*****n*** **=** **1]**		**The Hybrid Laboratory**
**Kost** *Med Lab Obs* 1992 (73) *Arch Path Lab Med* 1992 (74) *Crit Rev Clin Lab Sci* 1993 (75) *Prin Pract POCT* 2002 (76)		**The hybrid laboratory: shifting the focus to the point of care** (73) **The hybrid laboratory. The clinical laboratory of the 1990s is a synthesis of the old and the new** (74) **New whole blood analyzers and their impact on cardiac and critical care** (75) **The hybrid laboratory, therapeutic turnaround time, critical limits, performance maps, and Knowledge Optimization^®^** (76) Hallmarks of the hybrid laboratory are *distributed, but clinically integrated testing*, bedside and near-patient testing, customized test clusters, minimized TTAT, optimized temporal and Dx-Rx processes, the total quality principle, collaborative teamwork, increased productivity, and especially evidence-based medicine and improved outcomes, all important driving forces behind POCT. An essential principle is minimization of the time patients spend at high risk. POCT promotes cost-effectiveness because efficient diagnosis (Dx) and efficacious treatment (Rx) improve outcomes and spare resources. Collaborative teams apply POCT for Dx-Rx process optimization at the bedside. Therefore, the hybrid laboratory perpetually *shifts the focus to the point of care*.
**S12 [*****n*** **=** **1]**		**Point-of-Careology**
**Liu** *Point of Care* 2019 (77)	China and other world settings	**The Creation of Point-of-Careology** The objectives were to improve awareness of POCT as a new medical field, to solidify relationships among POC professionals, and to identify potential for advancing medical applications, economic benefits, and patient impact through timely decision making for evidence-based medicine. POCT now is being written into a professional textbook in medical schools in China. POCareology is the outcome of evolution in intelligent diagnostics encompassing all forms of POC technologies. Notable achievements in critical care medicine, emergency response, and general practice have resulted from the implementation of POCT over the past four decades. As a new discipline, POCareology will contribute to key medical areas, such as disaster preparedness and public health. The creation of this new specialty is justified by trends in modern medicine with improved service to the public and by parallel technological advances that empower health care providers at sites of need to deliver complete care cycles quickly and effectively.

## Introduction

### Rationale

Synthesis of geospatial and POC concepts can facilitate emergency care, crisis response, and control of highly infectious diseases, such as Ebola virus disease (“Ebola”). Integration of both concepts improves population access to healthcare. For efficiency and cost-effectiveness, POCT must fulfill healthcare needs on a daily basis, improve diagnostic skills, and enable public health, emergency medicine, and other personnel with fast decision making.

### Definitions

Point-of-care testing is defined as diagnostic testing at or near the site of patient care (78, 79). It is *inherently spatial*, that is, performed at or near points of need, and also *intrinsically temporal*, because it produces fast actionable results. This definition does not depend on the size or format of the handheld, portable, or transportable instrument, test module, or assay design.

It includes disposable test strips and *in situ, ex vivo, in vivo*, and *on vivo* monitoring (e.g., pulse oximeters or wearables). The “Cape Cod” group (80) codified the definition, which appears in standard dictionaries of the English language. The POCT•CTR wrote the original Wikipedia article (see https://en.wikipedia.org/wiki/Point-of-care_testing).

### Organization

Table 1 focuses on the geospatial tools most relevant for creating the right location and timing for implementing POCT, especially in resource-limited settings. For example, small-world networks (SWN) led to striking nation-wide improvements in the care of patients with acute coronary syndromes in Thailand (see Table 1, Solution 1). References cited in Table 1 provide practical details, so readers can custom tailor solutions to their own settings.

## Background

Highly infectious diseases, regional wars exacerbating deadly outbreaks [e.g., Ebola in the Democratic Republic of the Congo (DRC)], populations growing exponentially at least until mid-century (81), dense coastal communities, global warming and rising oceans, weather catastrophes, and migrating refugees seeking refuge and asylum, among others, challenge our ability to rectify health disparities. The Global Preparedness Monitoring Board (GPMB) warns, “The world is at acute risk for devastating regional or global disease epidemics or pandemics that not only cause loss of life but upend economies and create social chaos (82).”

However, the GPMB report did not recognize the importance of geospatial science and POCT. Population clusters in highly vulnerable areas amplify the risk. Nonetheless, we must eliminate inequities, while also responding quickly and effectively to increasingly frequent disasters, complex crises, and epidemics. To understand how to mitigate risk, we can use geospatial science mappings and produce “visual logistics,” that is, easily understood graphics, flowcharts, topography maps, time contours, and other illustrations that show where, when, and how to position POCT optimally, create mobile rapid response, and implement the solutions in Table 1.

## Methods and Scope

### Methods

PubMed, the World-wide Web, web sites of educational institutions, and other relevant sources were searched for papers, articles, chapters, documents, flowcharts, maps, and schematics in the form of published works and field research in limited-resource and other settings. Searches using IEEE Xplore did not yield additional relevant articles. In the interests of brevity, the reader can find detailed discussions of geospatial theory, software apps, and analytical techniques in Ferguson et al. (28–30). Visual logistics illustrate several key concepts. EndNote X9.1 (Clarivate Analytics, https://clarivate.com/) was used to consolidate over 500 entries retrieved as abstracts, ULRs, and PDFs, then subsequently pruned to slightly more than 100 articles to focus this review.

### Scope

Numerous sources identified through PubMed dealt with the general area of geographic information systems (GISs) in healthcare. The majority addressed GISs for tracking, monitoring, and managing infectious diseases, such as malaria and HIV. This article assesses the importance of geospatial science as it pertains specifically to POCT. Only those geospatially oriented publications explicitly discussing or integrating POCT and mobile technologies in relevant spatial settings, such as ring vaccination and space flight, are assessed here. Molecular diagnostics for Ebola and other highly infectious threats can be found in comprehensive reviews (36, 83) and a book chapter (44).

## Geospatial Science

This section analyzes 10 geospatial science approaches (Table 1) where optimal interplay of space and time can enhance healthcare and improve positioning of POCT resources. It also identifies current research gaps and future horizons. Historically, SWNs facilitated placement of POC cardiac biomarker testing in limited-resource emergency rooms of Thailand, so we start with that, then move through space-time transformations, spatial care paths™ (SCPs), geographic information systems (GISs), and other high yield solutions.

Outbreaks are spatially dynamic. Starting with the recent epidemic in 2014, stopping Ebola outbreaks from spreading and caring for infected patients who are critically ill unequivocally proved the need for POCT (16, 34–36, 44, 83). The current situation in the DRC is no exception. Hence, Table 1 also covers physical spatial designs needed to address safe handling of highly infectious threats, care for infected patients, and render communities more resilient.

## Small-World Networks and Regional Topography (Solution 1, Table 1)

**Figure 1:**
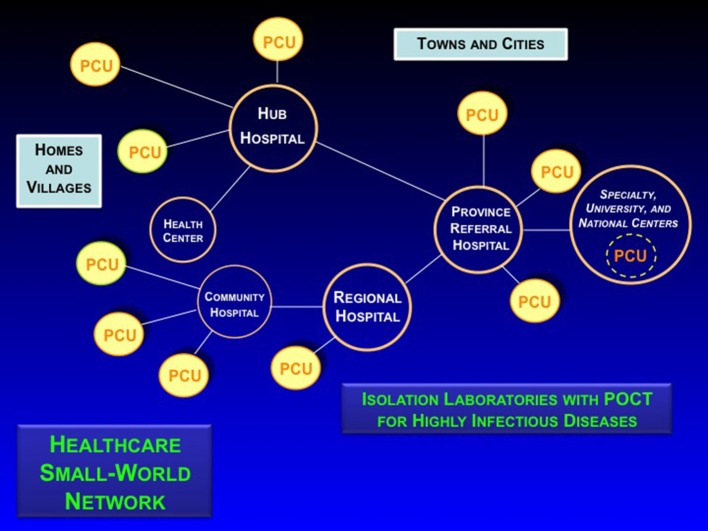
Small-world networks in limited-resource settings. Primary care units (“PCU”) serve patients close to home. Clusters, nodes, and relatively few connections characterize the typical healthcare SWN in a limited-resource country. Hub hospitals can expedite referrals to the province hospital. The SWN should include isolation laboratories for rapid and safe testing of specimens from patients with highly infectious diseases. Image reproduced with permission of Knowledge Optimization.

Small-world networks evolve naturally from social, political, and economic interactions; population dynamics; and medical-cultural ecosystems. Figure 1 illustrates relationships in a typical healthcare in a SWN in a limited-resource setting. Analysis of SWNs discloses how healthcare is delivered, whether population access is adequate, and where gaps in emergency service occur. Unique topographic features, such as mountains, lakes, and rivers within a limited geographic region typically bound a specific SWN. During a crisis, transportation routes can become constrained by storms, floods, earthquakes, interrupted roadways, telecommunication disruptions, systems failures, unexpected mishaps, supply chain shortages, and failures of emergency vehicles and aircraft. Rescue and response become compromised, complicated, and prolonged, often at the cost of human life.

Practice principles for optimizing POCT in SWNs are designed to enhance disaster preparedness, emergency management, and public health response at local (district, county, province, or state), regional, and national levels, while simultaneously improving urgent and routine care in the community. This allows POCT operators to garner substantial experience with the use of POC instruments, reagents, and quality assurance through daily experience. POCT should not be used without proper training of those who will perform testing and quality control.

For details of SWN theory, please see “Using small-world networks to optimize preparedness, response, and resilience,” chapter 49 in *Global Point of Care: Strategies for Disasters, Emergencies, and Public Health Resilience* (12). Briefly, a SWN represents a loosely tied and well, but not necessarily evenly, connected set of nodes and clusters in a scale-free network with a topology that is neither completely regular nor entirely random. Most nodes in a SWN are not neighbors, but can be reached in a few steps. Scale-free networks have hubs of connectivity that shape the way the network operates. Hubs provide robustness to failure, a key point when improving community resilience. Hubs connect nodes locally while also connecting clusters globally, conferring SWN properties to the healthcare system.

Regional hubs represent an advantage when planning POCT sites. However, removal of a busy hub during a disaster, because of physical destruction, invasive flooding, or some other calamity can turn the SWN into an isolated graph. Hence, resilience depends in part on self-reliance at individual nodes and clusters, an attribute that POCT confers nicely by enabling evidence-based decision making and local treatment. Only a few edges (interactions) separate nodes, a property leading to the popular notion of only 6 degrees of separation between any two people in the world. Separation impacts SWNs, so separation, *per se*, is to be avoided. Transit and information delays among SWNs can be fixed with physical splines (e.g., transportation “short-cuts”) and virtual connectivity (e.g., using POC disposable test modules on smartphones with built-in wireless communication).

**Figure 2:**
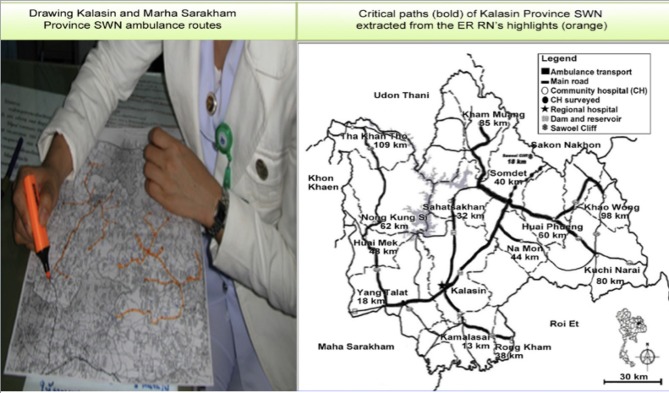
Configuring the healthcare small-world network, Kalasin Province, Thailand. Image reproduced with permission of Knowledge Optimization.

Small-world networks must be discovered, drawn, and metricized through grass roots field investigations and interviews of emergency personnel. Figure 2 illustrates the process of discovering the characteristics and infrastructure of a SWN in rural impoverished provinces in Isaan, Northeast Thailand. Interactions, especially emergency links, infectious vectors, and other systematic and random phenomena tend to transcend simple policy, roadmaps, phone lines, cellular towers, and radio transmissions. They encompass personal and professional interactions, resource limitations, adaptable social networks, and government requirements regulating tiered use of health maintenance sites, primary care units (PCUs), clinics, community and regional hospitals, and university or referral medical centers. Their interdependencies, both routine and emergent, often cross established boundaries and bridge international borders through fluxes of supplies, laborers, professional personnel, and also, communicable diseases.

**Solution 1** in Table 1 provides analysis (right column) of SWN field applications of POCT in limited-resource settings. Typically, these settings do not have helicopter or fixed-wing aircraft rescue and often lack adequate ambulance service. After successful pilot studies and educational seminars held for emergency staff, critical care nurses, and cardiologists, an executive decision was made at the national level by an academic (GK, while a Fulbright Scholar), professional, and industry team to implement approximately 700 POC cardiac troponin T (cTnT) handheld devices (cobas h 232, Roche Diagnostics) in over 500 community hospitals, revolutionizing the care of acute coronary syndrome patients in Thailand [**5**]. The other applications under **Solution 1** demonstrate similar utility and value of POCT in SWNs. The keys are flexibility and adaptability. Well-placed POCT can make SWN response “organic,” that is, rapidly adaptable in the face of evolving needs and crises.

## Space-Time Transformation, Hubs, and Asset Mapping (Solution 2)

**Figure 3:**
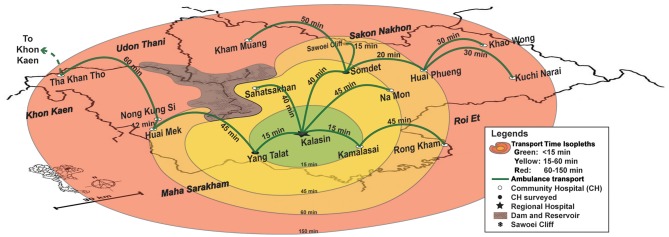
Transforming the small-world network from the physical to the temporal domain. Emergency room physicians in the Tha Khan Tho Community Hospital near the border of Kalasin Province (upper left) could refer acute coronary syndrome patients directly to an interventional cardiologist at the Khon Kaen Heart Center if elevated cTnT rules in acute myocardial infarction. Otherwise, patients are referred to the Kalasin Regional Hospital, but transportation is prolonged. At the time of the field survey, no cardiologists were available in the province, so patients would only receive enzyme or palliative treatment, unless referred to the Heart Center. Hence, POC cTn, rapid response, and efficient routing increase chances of survival. Image reproduced with permission of Knowledge Optimization.

Figure 3 illustrates space-time transformation of the healthcare network with analysis of mobility, geographic routes, and ambulance travel. For example, an emergency room physician in a community hospital distant from the provincial regional hospital could rule in the diagnosis of acute myocardial infarction using POC cTn testing, and then, save time by transporting the patient immediately and directly to a referral site where an interventional cardiologist is available (upper left, Figure 3), rather than routing the patient through the regional hospital in the center of the province when there is no cardiologist available.

**Figure 4:**
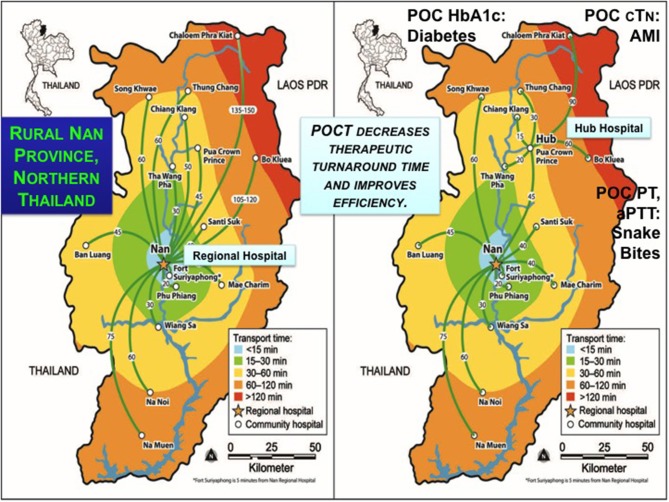
Contour maps and hub concept for efficient regional care in Nan Province, Northern Thailand. The left frame shows the SWN of rural Nan Province, and the right, the utility of a hub hospital to improve efficiency by decreasing transit times indicated by temporal contours. Pont-of-care testing can accelerate evidence-based decision making for the conditions shown, which community hospitals identified as high priority. Image reproduced with permission of Knowledge Optimization.

Figure 4 illustrates temporal contour map analysis in northern Nan Province of Thailand, where a team from the POCT•CTR, Siriraj Hospital (Mahidol University), and the College of Population Studies (Chulalongkorn University) conducted extensive field surveys of healthcare needs, resources, transportation routes, emergency medical systems/services (EMS), radio dispatching, and delivery gaps that could be filled with POC, near-patient, or satellite laboratory testing. The right frame shows the benefit of reduced response time when there is a regional hub. Hubs arise because combining resources overcomes constraints. Contour analysis clarified trade-offs of time and locale in the province SWN. The hub hospital substantially improved the efficiency of providing care to those living near the Thailand-Laos PDR border.

A surprise outcome in Nan Province was that nursing staff in the most northern regions adjacent to Laos PDR in Chaloem Phra Kiat Community Hospital insisted POCT be implemented to enable young physicians to make evidence-based decisions based on diagnostic bedside test results. Education became a primary motivator for implementing POCT. Junior physicians are conscripted to work in rural hospitals for 3 years to pay back government funding of their education. The number of people per doctor ranged from a low of 954 in Bangkok to a high of 8,510 in neglected resource-limited northern and northeastern provinces (64, 65). Often, the young doctors are on duty by themselves throughout the night without backup and no access to definitive diagnostic tests, such as the biomarkers of cardiac injury.

**Figure 5:**
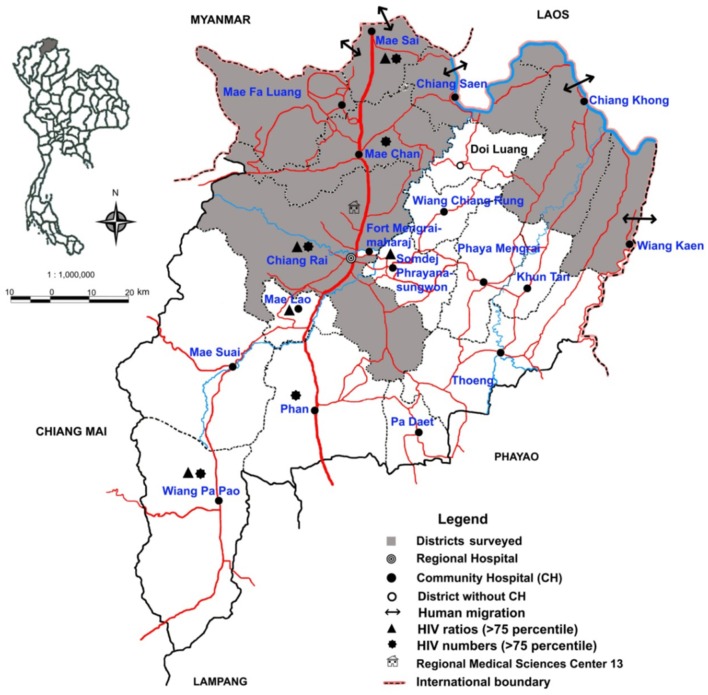
Chiang Rai Province small-world network and human migration across the Laotian border to Thailand and back. Clandestine and night crossings by boat on the Mekong River (blue line, northeast) allow prolific international human traffic into and out of the province. That brings HIV and other problems associated with migration to bear on the healthcare SWN. Clinic point-of-careologists could help abate spread of unreported disease. Image reproduced with permission of Knowledge Optimization.

During interviews the physicians said they did not like watching their patients, perhaps with equivocal EKGs, die while under observation, but instead wanted to use POC cTn testing to rule in acute myocardial infarction and refer quickly. Similar themes (see Figure 4) of rapid response cropped up for urgent POC coagulopathy tests (PT and aPTT) when children presented to the emergency room at high risk from potentially fatal snakebites and the only analysis available was timing blood clotting in a tube. For diabetes, medical staff wanted to use immediate onsite HbA1c results to avoid long specimen transport to referral sites, week or longer delays waiting for results, time and money consuming return trips by patients, and their forfeiture of employment income. HbA1c also can help identify patients with diabetes in field facilities, quarantine, and isolation for highly infectious diseases, such as Ebola when specimens cannot be sent to the clinical laboratory for fear of contamination. In settings where population migration complicates public health screening, such as influx of workers with HIV and possibly also TB into the SWN from across a natural geographic border (Figure 5), rapid response testing can facilitate patient workup and case reporting for epidemiological databases in primary care sites.

## Spatial and Geospatial Care Paths™ (Solution 3)

A spatial care path (SCP) is defined as the most efficient route taken by the patient when receiving definitive care in a SWN (15). A geospatial care path™ (GCP) is a second generation SCP that integrates demography and other population factors to respond quickly to crisis stress (21). **Solution 3** in Table 1 presents analysis of several applications of SCPs where the logic of their design is to identify the best routes for patient rescue and transport facilitated by shrewd placement of POCT for rapid diagnosis, triage, and treatment.

**Figure 6:**
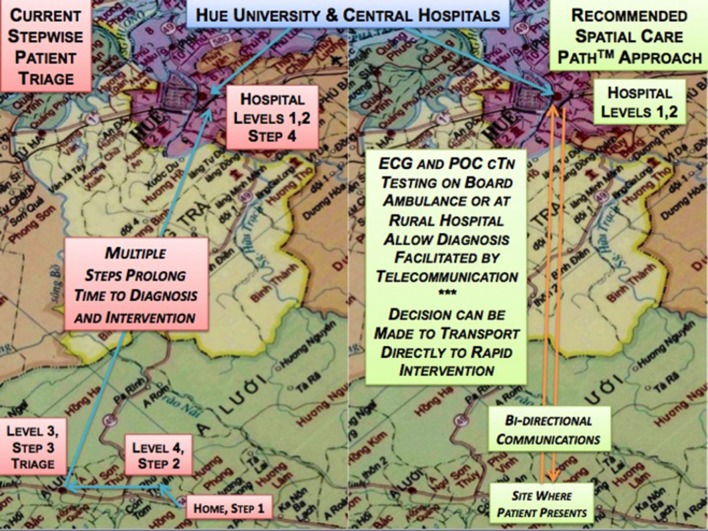
Spatial Care Path™ in Hue Province, Central Vietnam. Following an extensive needs assessment field survey of Hue Province, Central Vietnam, analysis of the traditional stepwise process (left) of patient evaluation in the rural mountainous areas and in multi-level government care sites downstream showed response time for patients with suspected acute myocardial infarction could be improved substantially by adopting POC cardiac biomarker testing upstream and more direct routes (right) to cardiologist intervention in Hue City. Image reproduced with permission of Knowledge Optimization.

Figure 6 illustrates a SCP solution created after an in-depth field survey of Hue Province in Central Vietnam (19), where the rural mountainous regions present significant challenges for rescuing rural patients with acute chest pain. The same logic was applied to infectious diseases and diabetes care; for example, see the analysis for Ventura et al. (20) in Table 1. Spatial care paths also provide clever opportunities for implementing emergency room and satellite laboratory diagnostic testing in SWNs.

It is likely that the SCP concept eventually will dominate healthcare delivery in limited-resource settings as populations expand, the number of elderly increase, and common sense, not to mention financial necessity, dictate that diagnosis and treatment must shift upstream nearer to the site of the patient's home in order to conserve resources, save time, and spare lives. As the availability of POC technologies expands and costs decrease through manufacturing efficiencies, countries with burgeoning population, such as China, India, Indonesia, and the Philippines, will find it easier to move initial diagnostic evaluation to primary sites.

In Indonesia and the Philippines, local independence of healthcare nodes, clusters, and hubs becomes essential. Time is of the essence. Transport to larger islands where referral hospitals are located is challenging. In fact, island nations are at risk of flooding displacing dense coastal populations. POCT can follow these migrations. Hospital directors stated that 80% of their community population should be diagnosed and ideally, treated, in primary care sites outside the hospital, in order to prevent saturation of emergency rooms that simply cannot handle huge numbers of patients and unexpected surges that show up for evaluation (e.g., during a seasonal Dengue hemorrhagic fever outbreak).

**Figure 7:**
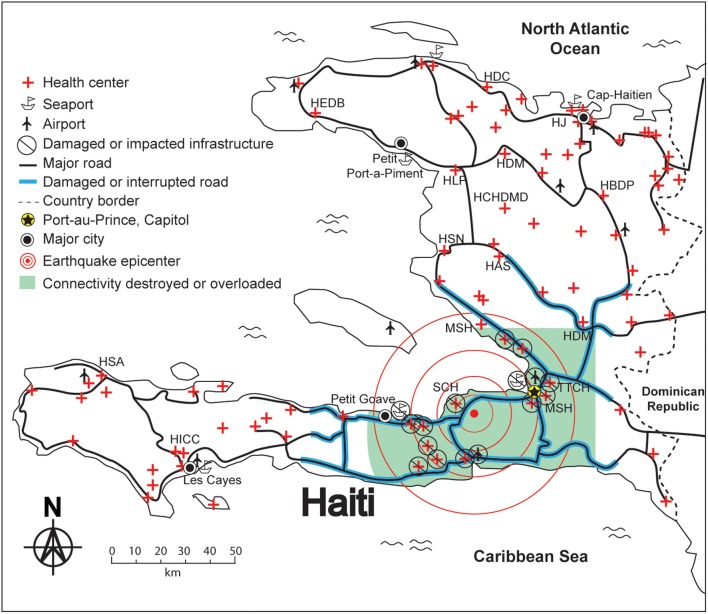
Geographic isolation by the Haiti Earthquake—need for spatial resilience. The circles show the earthquake epicenter. Bold lines show damaged or interrupted roads. United States Disaster Medical Assistance Teams carried suitcase size (<50 lbs) sets of POC diagnostics, but larger instruments transported to Haiti could not be used because there were no trained operators who could perform quality control. In settings like this one, communities should develop their own plans for resilience that include mobile POCT and properly trained, certified, and annually validated personnel. These resources should be placed at local nodes, clusters, or hubs in the SWN to assure optimal resilience. Image reproduced with permission of Knowledge Optimization.

Earthquakes in densely populated areas (Figure 7) warrant serious advance design and planning of SCPs to back up traditional routes to and from health centers and hospitals. With its numerous fault lines, fire hazard areas, limited rural transportation routes, and poorly maintained roads, a state like California should have a geospatial-POCT master plan to fill gaps in emergency management and preparedness. In other countries, the dynamic GCP concept can augment GISs analysis, especially in times of dire need, such as during volcano eruptions, tsunamis, and outbreaks of highly infectious disease.

## Geographic Information Systems (GIS) + Point-of-care Testing (POCT)

**Figure 8:**
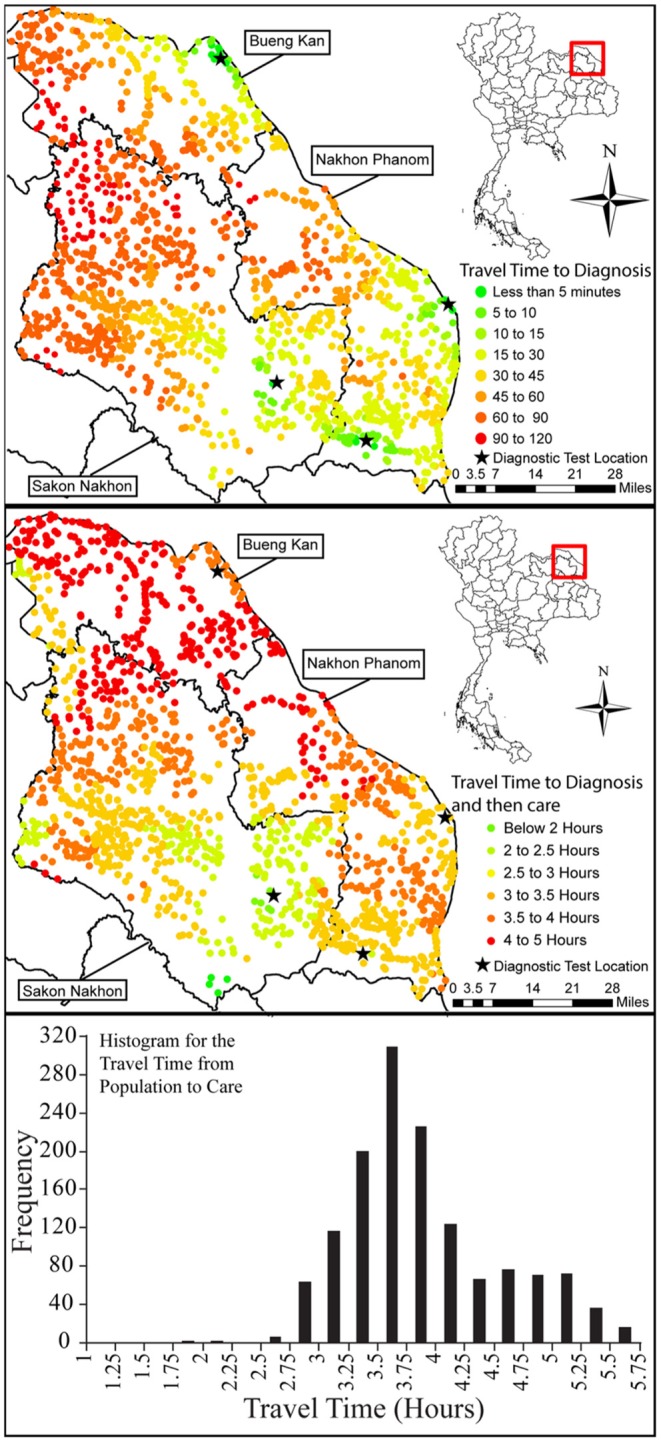
Travel times assessed using a geographic information system in the northern most region of Isaan, Thailand. Image reproduced with permission of Knowledge Optimization.

A GIS is designed to capture, store, manipulate, analyze, manage, optimize, and present a broad spectrum of geographical data relevant to the task at hand. While the healthcare literature is replete with GIS papers, some proving efficacy for highly infectious diseases, such as Ebola (84–87), **Solution 4** lists only those that combined both POC and GIS concepts, especially the analysis of population access in terms of travel time (Figure 8), an important criteria for deciding where to place POCT.

**Figure 9:**
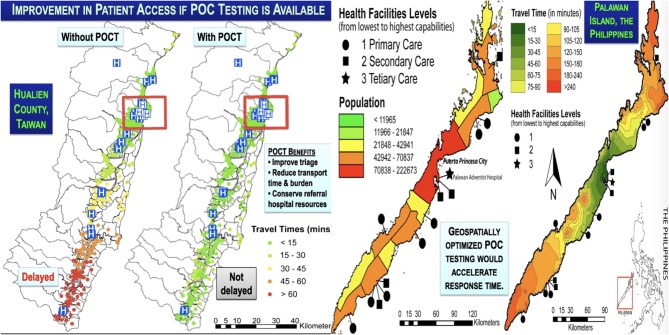
Geographic information system analysis of the impact of POCT in Hualien County, Taiwan, and Palawan Island, Philippines. Geographic information system analysis of population clusters illustrate how POCT could expedite patient access to care in Hualien County, Coastal East Taiwan (left), an area frequented by devastating typhoons. In the Philippines, extremely remote Palawan Island (right) is challenged by a similar long linear topography with poorly accessible rural northern and southern regions that lend themselves to geospatially placed POCT to improve patient access to healthcare. Image reproduced with permission of Knowledge Optimization.

For example, Figure 9 illustrates GIS determination of how POCT could expedite patient access to care, triage, and intervention in high risk coastal Hualien County, Taiwan, and on remote Palawan Island, the Philippines. GISs are also useful for analyzing patterns of spread of infectious diseases and dynamically interactive treatment, such as ring vaccination (a geospatial strategy to inhibit spread by vaccinating those most likely to be infected).

**Solution 4** entries illustrate how to integrate POCT as an element of public health strategy. For example, spatial grids enabled with POCT can locate sentinel cases and establish geographic limits of epidemics. To encourage these types of creative strategies, curricula of public health educational institutions should include training in the principles and practice of POCT (88, 89). As noted earlier, education represents an adequate reason for implementing POCT.

## Isolation Laboratories, Diagnostics Isolators, and Mobile Laboratories for Highly Infectious Diseases (Solution 5)

The 2014–16 Ebola epidemic proved unequivocally the need for POCT in isolation laboratories and diagnostics isolators to support critically ill patients in isolation (16, 34–36, 44, 83). These solutions are *distinctively and discretely spatial*. Part of public health repertoire, they should be incorporated into the scheme of the typical SWN, as recommended in Figure 1.

**Figure 10:**
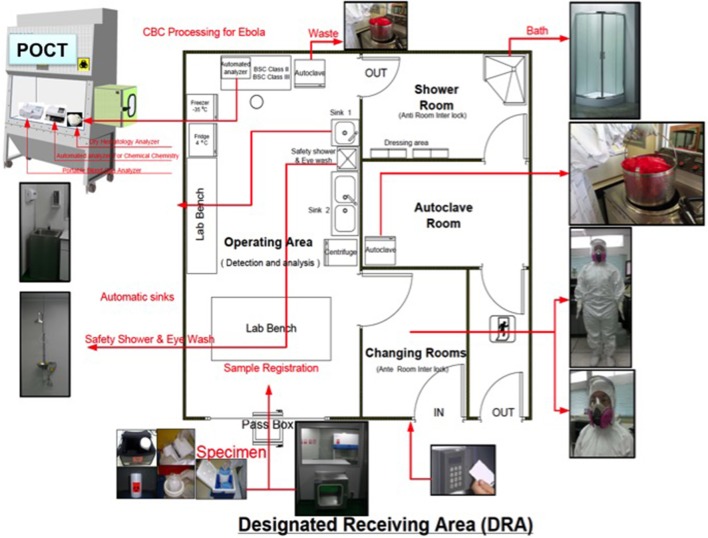
Design of an isolation laboratory with POCT inside a Biosafety Cabinet. This discrete spatial solution, an isolation laboratory design built in several Bangkok locations, offers vital engineering controls that reduce the risk of biohazards while also providing environmental temperature and humidity conditions for the proper operation of POC instruments. Personnel must practice donning personal protective equipment (PPE), then performing testing, followed by careful doffing, training that now is being implemented in US sites, such as the Center for Disaster Medicine at Massachusetts General Hospital (see https://www.massgeneral.org/News/newsarticle.aspx?id=7175). Image reproduced with permission of Knowledge Optimization.

**Solution 5** references (see Table 1) provide details of diagnostics isolators and isolation laboratories built and completed in response to the 2014–16 crisis in West Africa and the spread of Ebola to other continents. Figure 10 provides a schematic and workflow diagram of our design built in several hospitals in Bangkok. Chapter 24 (44) of *A Practical Guide to Global Point-of-Care Testing* presents isolation laboratories with POCT placed inside biosafety cabinets (16, 35, 36) and conceptual designs for safe self-testing (“FAST•POC”) and assisted testing (“POC•POD”). Types of instruments, diagnostic tests, and isolator designs can be found in Kost et al. (16, 34–37, 44, 83).

Not all tests that have been used in isolation laboratories, such as prothrombin time (PT) (34), are US FDA-cleared for Ebola patients. A follow-up interview in 2017 describing the Emory Serious Communicable Disease Unit (SCDU) enumerated tests as: “Ebola virus PCR results were available in about 1.5 h after receipt of the specimen in the SCDU laboratory. Other tests available included CBC, CMP, magnesium, lactate dehydrogenase, gamma-glutamyl transferase, amylase, lactate, phosphorous, venous/arterial blood gases, urinalysis, FilmArray^®^ gastrointestinal panel, FilmArray^®^ respiratory panel, BinaxNOW^®^ malaria assay, and Alere DetermineTM HIV-1/2 Ag/Ab Combo test (90).”

Kost et al. (36, 83) summarized molecular diagnostic technologies that emerged during and after the Ebola crisis. Food and Drug Administration Emergency Use Authorization (EUA) technologies can be found here: https://www.fda.gov/emergency-preparedness-and-response/mcm-legal-regulatory-and-policy-framework/emergency-use-authorization#current, and World Health Organization Emergency Use and Assessment Listings, here: https://www.who.int/diagnostics_laboratory/eual/emergency/en/.

Outbreaks continue in the DRC, now declared a global health emergency by the WHO. The number of cases (as of October 2019) totaled 3,274 with a death toll of 2,185 and mortality of 67% (see https://www.who.int/emergencies/diseases/ebola/drc-2019/). This Ebola outbreak is the second worst, in the face of a response marred by war zones, shootings of health workers, civil strife, abandonment by NGOs and healthcare personnel, strike threats by nurses, and resistance within local communities to preventative measures, care facilities, and safe burials. Several care centers (>132) and health care workers (300+) have been attacked. Nonetheless, novel POC technologies, when used with safe specimen processing, can enhance ring vaccination, which has benefited the DRC by mitigating spread.

On February 26, 2019, the CDC, FDA, and CMS announced a new “Tri-Agency Task Force for Emergency Diagnostics (TTFED),” (http://www.fda.gov/NewsEvents/Newsroom/PressAnnouncements/ucm632056.htm). The charter can be found here: http://www.fda.gov/downloads/EmergencyPreparedness/Counterterrorism/MedicalCountermeasures/MCMLegalRegulatoryandPolicyFramework/UCM631575.pdf. The consortium stated, “Through the TTFED, CDC, FDA, and CMS, where appropriate, intend to coordinate the implementation of EUA IVD assays in laboratories within the U.S. healthcare system, with the ultimate goal of improving responses to public health emergencies.” However, there is no task force plan to train public health students or POCT specialists in the use of EUA devices and associated quality control. Except for one medical technologist, laboratory medicine professionals, public health educational institutions, and industries developing new EUA technologies appear not to be represented.

The TTFED's focus on EUA IVD assays falls short of the need for strategically selected POC technologies that integrate and consolidate a broad range of tests intended to help the Ebola patient with a highly infectious disease survive. Samples cannot be sent to the clinical laboratory. If spilled or broken, the clinical laboratory must be shut down for clean up, causing unacceptable delays in hospital services.

Well-integrated and compact POC technologies with comprehensive test clusters are needed to fit within the confines of isolation laboratories and diagnostics isolators, which represent uniquely discrete spatial solutions. The devices must be user friendly for operators suited in personal protective equipment (PPE). Environmental conditions must be controlled, not only to assure accurate test results, but also for the comfort of operators wearing PPE.

## Alternate Care Facilities; Roaming POCT—Airborne, Ambulances, Space, and Wearables; Connected and Wireless POCT Outside Hospitals; and Unmanned Aerial Vehicles (UAV, Drones) (Solutions 6–9)

**Figure 11:**
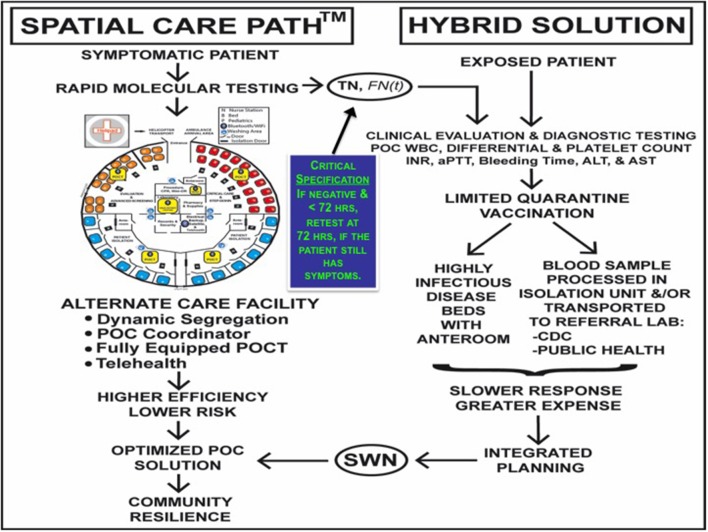
Spatiotemporal approach to highly infectious diseases with alternate care facility. This GCP design incorporates immediate high sensitivity POC molecular diagnostic testing to rule out the highly infectious disease, with the caveat that the false negative rate is a function of time and may be high initially when the viral load is evolving, requiring repeat testing. The alternate care facility (left), which is replete with POCT, provides a discrete spatial solution that is safe for patients, healthcare personnel, and the community. Screening, hospital quarantine, and specimen transport to distant referral laboratories for diagnosis (right) slow down patient throughput. Image reproduced with permission of Knowledge Optimization.

**Solutions 6–9** summarize spatially discrete solutions outside the hospital. Alternate care facilities (**Solution 6**) provide safe sites for quarantine and screening (Figure 11). Roaming POCT (**Solution 7**) will benefit from progressively smaller, smarter, and faster POC technologies adapted for remote applications, such as airborne (45–49), ambulances (50, 51, 91), space (52–55, 92), and wearables (52–55, 93, 94). Cummins et al. (95) reviewed POC technologies suitable or potentially usable in the micro-gravity international space station, during space flight, and possibly within confined space colonies subject to different gravitational fields (52).

Regardless of where they are generated, test results must be connected (**Solution 8**) for seamless bidirectional electronic communications. Physical linkages using novel unmanned aerial vehicles (UAVs) (**Solution 9**) show promise for delivery of critical medical supplies, transport of laboratory samples, and spatial solutions for accessing challenging topographies, remote sites, and isolated islands. In limited-resource settings, drones can help optimize healthcare SWNs, document the status of disasters, and track outbreaks of highly infectious diseases.

Pilot studies show feasibility of drones for specimen transport (59–62) and instruments for rapid pathogen detection during flight (63). Zipline (https://flyzipline.com/) UAVs supply blood, vaccines, and drugs throughout Ghana and Rwanda in Africa. They claim 500 deliveries per day serving 11 million, with 30 min response time from app-initiated order. The drones have a 1.8 kg payload, fly 40,000 km per week (cumulatively over 1 million km), launch at 100 km/h, drop the payload at the target, and then return to base. This system could transport POC devices, reagents, and QC materials to remote sites within the 80 km service radii of base stations, which now cover most of Rwanda and central Ghana.

## Geospatial Practice—Demographic Care Units, Geographic Risk Assessment, and National POCT Policy and Guidelines (Solution 10)

### 10.A. Demographic Care Units

The demographic care unit (DCU) concept gives policy makers a means of identifying the geographic locations most in need of POCT to improve standards of care (64, 65). DCU scoring can be applied to any country on a periodic basis to help identify inequities in regions of highest need. Thai provincial demographic features, health resources, and poverty measures were obtained through web research, published documents, and data from the MOPH and Office of the National Economic and Social Development Board. The number of people per individual health resource in each Thai province, that is, in each DCU, was, calculated utilizing the mid-year population.

Health resource characteristics comprised the number of people per primary care unit, hospital bed, medical doctor, registered nurse, technical nurse, pharmacist, and medical technologist in a given province. The total number of resource categories above the cut-off was determined to obtain the score for each DCU. Summary statistical analyses included range, minimum, maximum, mean, median, percentile, and standard deviation. The seventy-fifth percentile of people per health resource in a province defined the cut-off for interpretation of whether a DCU qualified as having a health resource deficiency. Also reported are poverty lines, number of poor people, poor people ratio, poor people times health resource score, and death rates.

**Figure 12:**
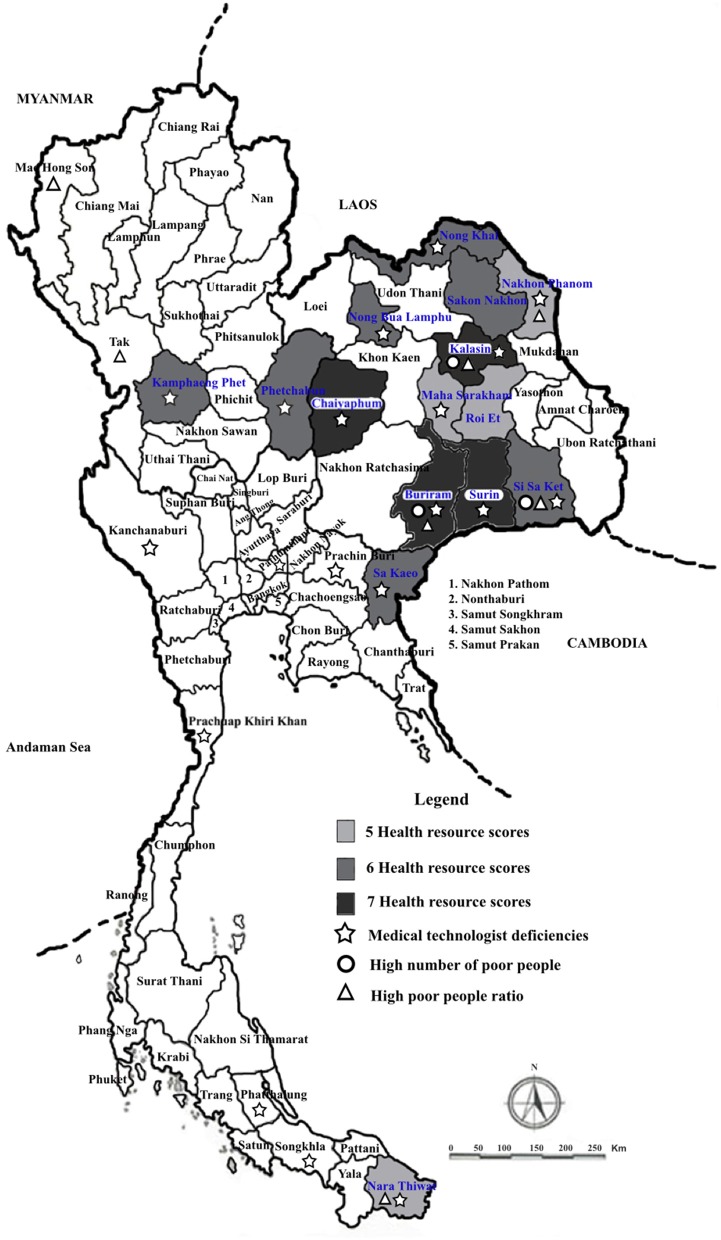
Demographic care unit scoring for Thailand. Image reproduced with permission of Knowledge Optimization.

The higher the score, the more inadequate the health resource was relative to the number of people served. Figure 12 shows the striking geographic inequities in healthcare resources, the most deficient located mainly in The Northeast (Isaan), where most DCU scores were 5, 6, or 7. One advantage of this approach lies in its ability to use current data to update priorities for the geographic placement of POCT. Medical technologists who operate POC technologies should accompany POCT placements. Combined DCU and SWN analysis (see **Solution 1**), educational programs, a Thai language book with chapters on POCT (96, 97), and a collaborative strategy implemented by cardiologists, academicians, and industry significantly improved the standard of care for acute coronary syndrome patients, as described earlier.

### 10.B. Geographic Risk Assessment

**Figure 13:**
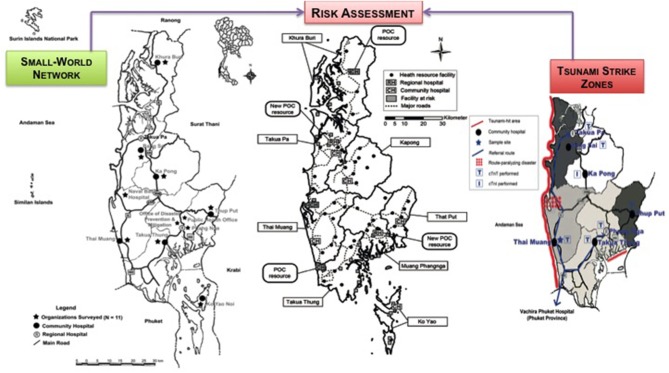
Risk assessment in Phang Nga Province hit by the massive Andaman Sea Tsunami. Image reproduced with permission of Knowledge Optimization.

The Southern Thailand case study in Figure 13 illustrates how to identify high risk in coastal SWNs. The map on the left shows the Phang Nga Province SWN, and on the right, Khao Lak and other areas hit hardest (in red) by the tsunami generated during the 2004 Andaman (Indian) Sea Earthquake. The center identifies hospitals and healthcare resources at risk. The earthquake was the third largest ever recorded. Waves as high as 30 meters smashed the coastal areas of Phang Nga Province and flooded the interior. The right panel shows blocked roads and routes to alternate hospitals.

While one account touted successful disaster response at the Takuapa District Hospital slightly north and inland in Phang Nga Province (98), several others more objectively documented broad failure and inadequate preparedness in community hospitals, especially coastal resorts and muubaans (villages) hit by the tsunami (67, 68, 99, 100). For example, the care of critical patients was compromised by lack of blood gas instruments throughout all community hospitals, and at the time, an analyzer that performs blood gases (pO_2_, pCO_2_), pH, and electrolyte (e.g., K^+^, Na^+^, and Ca^+2^) tests simultaneously on whole blood was inoperable in the Takuapa District Hospital, to which hundreds of critically injured tsunami victims were transferred (67, 68).

We (GK with three Chulalongkorn demography and economics graduate students on field survey) talked with the only doctor on call at one of the small coastal community hospitals in Phang Nga when the tsunami hit—roads were blocked by flood waters isolating the hospital, drugs were depleted rapidly as he triaged over 1,000 victims, diagnostic instruments were inaccessible, and staff personnel were totally inadequate, because they could not reach the hospital at the time of the crisis. The experience had a devastating psychological effect on the sole young physician in charge, who did not want to continue medical practice subsequently.

During follow-up surveys several years after the tsunami, still no blood gas testing (pO_2_, pCO_2_, pH) was found in any community hospital in Phang Nga Province (68). Default use of fingertip pulse oximeters (small battery-powered O_2_ saturation monitors, see https://www.youtube.com/watch?v=9ELSR7z0U4w) increased after the tsunami, but was complicated by the presence of several different brands that had been donated. In spite of dire need, the frequency of use of pulse oximeters in Phang Nga community hospitals did not match that in a survey control province, Chiang Rai, in northern Thailand (68). Detailed field studies are needed to fully assess which point-of-need diagnostic capabilities remain absent or at risk because of reagent supply chain issues, and whether, in fact, communities and ministries of health have acted responsibly to prepare for potential future disasters, such as a repeat tsunami in Southern Thailand or Indonesia.

In view of high probability of future occurrences of tsunamis from “ring of fire” earthquakes exacerbated by global warming and rising ocean levels encroaching on shore areas, geographic risk assessment should be performed in vulnerable coastal settings, particularly those with dense native population clusters and high tourist capacities. Point-of-care testing results are immediate and can be deployed rationally to support decision making based on diagnostic evidence obtained at points of need.

**Figure 14:**
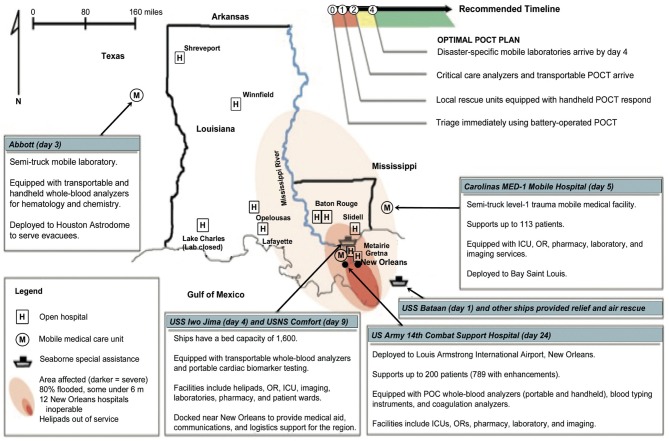
Geographic Risk—Delayed response during Hurricane Katrina in the US. The inset (upper right) suggests how the response timeline could have been improved using POCT resources. Image reproduced with permission of Knowledge Optimization.

Similar delayed and inadequate response occurred during Hurricane Katrina in the United States (1), because of the complexities of the coastal topography, severe flooding, and submerged metropolitan areas (Figure 14). Knowing patient status and diagnosis rapidly on site can speed response and allay anxiety, so medical staff can endure the nearly impossible stress, not to mention save lives and mitigate economic and cultural losses. Following Superstorm Sandy, the largest Atlantic hurricane on record, which flooded several basement clinical laboratories in New York City, United Healthcare Workers recognized POCT as the first line of defense for emergency preparedness and risk mitigation (101), but public health agencies have not followed up adequately.

### 10.C. National POCT Policy and Guidelines

A Malaysian national steering committee led by Dr. Baizurah produced the world's first national POCT policy and guidelines (71, 72), which was published in the English language. Please access the document directly (at http://www.moh.gov.my/moh/images/gallery/Garispanduan/National_Point_of_Care_Testing.pdf) to appreciate and understand the scope, depth, and breadth needed to harmonize POCT across the geopolitical expanse of the South China and Natuna Seas that separate mainland Malaysia and two of its states on the island of Borneo, a distance >1,000 miles (1,609 kilometers). The Malaysian initiative bases its guidelines on ISO (International Organization for Standardization) documents. Recognizing the importance of geopolicy in the use of new POC technologies can accelerate implementation, funding, and acceptance. Our book chapter (72) summarizes the status of other nation state-oriented policy and guidelines worldwide.

## The Hybrid Laboratory and Point-of-Careology (Solutions 11 and 12)

**Solutions 11** and **12** chronicle nearly three decades of spatial movement of whole-blood analysis to the bedside in the OR and ICU, known as the “*hybrid laboratory”* (73–76), progressing to the present day, when POC culture (11, 15, 18) has emerged as a new medical specialty, “*point-of-careology”*, in China (21, 77). The point-of-careologist performs testing and uses diagnostic results directly where he or she encounters patients in daily and emergency practice. Point-of-careologists also will fulfill multifunctional needs in confined spatial environments, such as the Mars colony and during extended extraterrestrial voyages. Physician astronauts (102) should be trained well in POCT and its quality assurance to anticipate this future.

## Conclusions and Limitations

### Critical Space and Time

POCT is *inherently spatial*, that is, performed at or near points of need, and *intrinsically temporal*, thereby accelerating critical evidence-based decisions. Point-of-care technologies have progressed well beyond mobile diagnostics to become a means for spatially and temporally optimizing preparedness, emergency management, and outbreak control. Advanced mobile technologies, such as modular assays attached to smartphones, will permeate healthcare.

However, without strategic planning, poorly organized SWNs will still suffer from disparities of access, service, rescue, response, and resilience. Geospatial care paths with integration of POC and other tests (103) selected to meet demographic priorities (64, 65) can fine-tune SWN logistics to accelerate point-of-need response and decision making. Additionally, parallel knowledge of geospatial distributions of diseases can aid the development, selection, and placement of POC tests specific for populations at risk.

### Resource Constraints

Increasingly, resources appear inadequate to meet the needs of future world population growing by 83 million people each year and expected to hit 10 billion by 2050. Demographers debate whether this mid-century world population will expand or contract (81). Either pathway spells trouble for healthcare, including island nations facing the threat of inundation of coastal communities because of global warming. Economic reserves likely will shrink absolutely or relatively with respect to these overwhelming burdens, diminishing access and exacerbating healthcare disparities. Point-of-need testing and geospatial innovations have the potential to alleviate these imbalances.

### Strategic Capacity

Surveys of public health curricula (88, 89) identified root cause deficiencies of POCT curriculum and geospatial education in schools and programs of public health. Inadequate professional training means that the workforce of POC specialists and geospatial scientists will not meet future needs. This deficiency must be corrected (88, 89, 104). Education of public health professionals in the principles and practice of *POCT* (78, 79) should start early, in order to generate trained, inspired, and enabled personnel who can make immediate evidence-based decisions in the field during crises and at community care sites.

### Geospatial·POC

Disasters, outbreaks, complex crises, climate warming, terrorism, and rampant shootings have emerged as global norms. With over seven billion people in the world, highly clustered communities, dense collections of inhabitants, and numerous populations at risk, the overall goal is to integrate principles and practice of POCT and initiate a fresh approach to public health preparedness and community resilience (105, 106). This applies to limited-resource and advanced settings alike, such as urgent care on the islands of Indonesia and weather disasters like the Great Bangkok Flood (66) in large metropolitan areas.

Since 1980 in the US alone, 246 disasters have exceeded $1 billion in costs, totaling $1.6 trillion (107). Substantial human, economic, and social losses will continue to burden the global community. Responding rapidly will save lives, valuable resources, and irreplaceable culture. Therefore, *geospatial*·*POC preparedness should become the new norm*. In fact, two recent books point us toward preparedness (108, 109), but omit POC technologies, geospatial science, and integrated time-space solutions. The building blocks in Table 1 used in conjunction with complementary educational curricula (88, 89) will fill these gaps and enable rapid response solutions at points of need worldwide.

## Public Health Recommendations

Public health professionals appreciate the importance of GISs and visualized health data in determining inequalities and inaccessibility issues and in addressing infectious disease epidemiology (110, 111). Geospatial solutions include isolation laboratories and diagnostics isolators for highly infectious diseases, but limited availability of this vital critical care support puts population clusters at risk (90, 112, 113). Therefore, key principles and value propositions for future implementation of geospatial science and POCT include—

- Educators in emergency management and preparedness should teach geospatial science and POCT principles, so that interventional public health practitioners will understand rapid response, point-of-need diagnostics, quality assurance, immediate decision making, isolation for critical treatment, and a fundamental need—spatial and temporal resilience.
- Spatially isolated laboratories and diagnostics isolators for the support of critically ill patients with highly infectious diseases, such as Ebola, can be positioned geographically to serve regional population clusters. These resources are distinctively and uniquely *spatial*, since they define biohazard “safe houses.”
- One can optimize these facilities by assessing travel, exposure (e.g., airline passengers), and treatment options in advance of outbreaks and epidemics, then equipping the facilities properly with POCT and personnel experienced in testing while wearing PPE, so they can operate instruments independently while in isolation.
- POCT-enabled spatial grids can locate sentinel cases and establish geographic limits of outbreaks. Integrating POCT plus GIS with ring vaccination will enable unique synergism for quickly detecting sentinel cases, geospatial tracking, and containment of epidemics.
- Analysis of population clusters, hubs, topographic features, and EMS routes in SWNs helps determine best sties for POC diagnostics, including devices on ambulance and other rescue vehicles or aircraft. These technologies also are needed for secure space flight.
- Small-world networks with multidimensional connectivity facilitate efficient and effective placement of POCT, which improves response time, rescue, diagnosis, treatment, and spatial resilience by accelerating evidence-based decision making at points of need.
- Visual logistics help design delivery systems capable of efficiently addressing individual crises, such as acute myocardial infarction, while analyzing healthcare strategies for difficult public health problems, such as Ebola, MERS-CoV, and H7N9; HIV, TB, and malaria; and differentiating concurrent Ebola infections from the others during outbreaks.
- Documenting transport time in contour maps and GISs facilitates EMS radio dispatching, regional coverage, and emergency transport, especially in the absence of helicopter transport or when ambulance service is inadequate.
- Placement of POC devices, ECG, and diagnostic tests on board ambulances with physician-guided prehospital diagnosis by telemetry accelerates pathways to tertiary care and intervention.
- Space-time transformations can identify POCT-facilitated (e.g., AMI ruled in by positive POC cTn testing) transport “short-cuts” for critically ill patients to referral centers for cardiologist intervention. Parallel analysis produces solutions for diabetes and other public health challenges.
- Use of GISs to position POCT close to where patients live and work facilitates triage and rapid therapeutic turnaround time during emergencies, disasters, and outbreaks. They can expedite urgent primary care and assist remote population clusters in need of access.
- Spatial care paths™ with POC devices positioned cleverly upstream speed transport from primary care to referral centers by bypassing geographic bottlenecks and time-consuming interruptions (e.g., roads flooded by seasonal downpours).
- Geospatial care paths™ encompass features of population clusters, demographic fluxes, and local POCT hubs to decrease therapeutic turnaround time by expediting intermediate stages of inefficient routines, government protocols, or perfunctory patient evaluations.
- Geographic information systems can provide backbones for these care paths and also for dispatching drones, routing flight paths, and delivering medical and POC supplies in remote or inaccessible areas.
- Analysis of demographic care units can guide ministries of health in providing adequate primary care access, hospital beds, physicians, nurses, pharmacists, medical technologists, POC coordinators, POC technologies, and other vital resources, especially when prioritizing rural regions most in need using fresh data renewed each year.
- Onsite, rapid, and highly sensitive/specific POCT will help identify highly infectious diseases, stop outbreaks, and establish topographic perimeters of contagion. False negatives as a function of time, *FN(t)*, must be minimized early on by assuring high sensitivity for ruling out infection of exposed patients.
- Alternate care facilities equipped with POCT can enhance community resilience in the event of quarantine, disasters, or complex crises (e.g., the earthquake-tsunami-radiation leak on coastal Japan).

## Closing Insights

### High Impact

The POCT industry has grown to $31.4 billion worldwide, projected at $37.0 billion in 2021 (114). The POCT growth rate in the Asia-Pacific, the region of several field research reports in Table 1, is 14.2%. Numerous POC technologies in the pipeline soon will emerge. For example, NIH-BARDA will award a $20 million prize, the largest in United States history, for POC inventions addressing antimicrobial resistance (see https://dpcpsi.nih.gov/AMRChallenge). The idea is to administer targeted therapy immediately to individual patients, avoid indiscriminate use of antibiotics in the population at large, and save precious healthcare funds.

### Future Direction

Best use of POCT occurs when shrewdly selecting, combining, and integrating solutions in Table 1 to create value, so that patient care is shifted toward the home, primary care, and community; critical care becomes more efficient; and deaths are avoided during public health crises. The education of young physicians in evidence-based medicine when delivering rapid emergency care in isolated remote settings constitutes adequate indication for implementing POCT. Inventions, innovations, and economies of scale will reduce POC manufacturing, supply chain, and utilization costs. In China the new medical field called “point-of-careology” (77) integrates diagnostics for rapid decision making at points of need by medical specialists enabled with POC technologies. This symbiosis will benefit professionals in other resource-, population-, and topography-challenged settings, such as countries with vast geographies and island nations, to care equitably for burgeoning populations.

### Standards of Care

If public health professionals, geospatial scientists, and POCT specialists join efforts to use the solutions enumerated in Table 1, collaborative teamwork can create resilient and equitable healthcare in the community at points of critical need during emergencies, outbreaks, epidemics, and disasters, as well as more efficient primary, urgent, and emergency community care. Importantly, they will improve public health and crisis standards of care.

## Author Contributions

The author confirms being the sole contributor of this work and has approved it for publication.

### Conflict of Interest

The author declares that the research was conducted in the absence of any commercial or financial relationships that could be construed as a potential conflict of interest.

## References

[1] Kost GJ Tran NK Tuntideelert M Kulrattanamaneeporn S Peungposop N. Katrina, the tsunami, and point-of-care testing: optimizing rapid response diagnosis in disasters. Am J Clin Pathol. (2006) 126:513–20. 10.1309/NWU5E6T0L4PFCBD9 16938656

[2] Kost GJ Newdemics, public health, small-world networks, and point-of-care testing. Point Care. (2006) 5:138–44.

[3] Kost GJ Suwanyangyuen A Kulrattanamaneeporn S The hill tribes of Thailand: synergistic health care through point-of-care testing, small-world networks, and nodally flexile telemedicine. Point Care. (2006) 5:199–204. 10.1097/01.poc.0000232580.36762.68

[4] Guandlapalli A Ma X Stat M Benuzillo J Pettty W Greenberg R Social network analyses of patient-healthcare worker interactions: implications for disease transmission. AMIA Symp Proc. (2009) 213–17. PMC2815400 20351852

[5] Kost GJ Kost LE Suwanyangyuen A Cheema SK Corbin C Sumner S. Emergency cardiac biomarkers and point-of-care testing: optimizing acute coronary syndrome care using small-world networks in rural settings. Point Care. (2010) 9:53–64. 10.1097/POC.0b013e3181d9d45c 20577572 PMC2888163

[6] Yu JN Brock TK Mecozzi DM Tran NK Kost GJ. Future connectivity for disaster and emergency point of care. Point Care. (2010) 9:185–92. 10.1097/POC.0b013e3181fc95ee 21547239 PMC3086779

[7] Kost GJ Kanoksilp A Mecozzi DM Sonu R Curtis C Yu JN Point-of-need hemoglobin A1c for evidence-based diabetes care in rural small-world networks: Khumuang Community Hospital, Buriram, Thailand. Point Care. (2011) 10:28–33. 10.1097/POC.0b013e3182078402

[8] Kleczkowski A Oles K Gudowska-Nowak E Gilligan CA. Searching for the most cost-effective strategy for controlling epidemics spreading on regular and small-world networks. R Soc Interface. (2012) 9:158–69. 10.1098/rsif.2011.0216 21653570 PMC3223629

[9] Kost GJ Theory, principles, and practice of optimizing point-of-care small-world networks. Point Care. (2012) 11:96–101. 10.1097/POC.0b013e31825a25b5

[10] Kost GJ Katip P Tangchit T Human immunodeficiency virus, population dynamics, and rapid strategies for medical diagnosis in the northern most province of Thailand—Chiang Rai. J Demography. (2012) 28:37–63.

[11] Kost GJ Katip P Vanasith K Negash H The final frontier for point of care: performance, resilience, and culture. Point Care. (2013) 12:1–8. 10.1097/POC.0b013e318266b7fe

[12] Kost GJ Chapter 49: using small-world networks to optimize preparedness, response, and resilience. In: Kost GJ Curtis CM editors. Global Point of Care: Strategies for Disasters, Emergencies, and Public Health Preparedness. Washington, DC: AACC Press-Elsevier (2015). p. 539–68.

[13] Girdwood SJ Nichols BE Moyo C Crompton T Chimhamhiwa D Rosen S. Optimizing viral load testing access for the last mile: geospatial cost model for point of care instrument placement. PLoS ONE. (2019) 14:e0221586. 10.1371/journal.pone.0221586 31449559 PMC6709899

[14] Mackenzie SLC Hinchey DM Cornfortrh KP. A public health service-learning capstone: ideal for students, academia and community. Front Public Health. (2019) 7:1–7. 10.3389/fpubh.2019.00010 30761285 PMC6361775

[15] Kost GJ Ferguson WJ Kost LE Principles of point of care culture, the spatial care path™, and enabling community and global resilience. e-Journal IFCC. (2014) 25:134–53. PMC4975289 27683461

[16] Kost GJ Ferguson WJ Hoe J Truong A-T Banpavichit A Kongpila S. The Ebola Spatial Care Path™: accelerating point-of-care diagnosis, decision making, and community resilience in outbreaks. Am J Disaster Med. (2015) 10:121–43. 10.5055/ajdm.2015.0196 26312494

[17] Kost GJ Ferguson WJ Spatial Care Paths™ strengthen links in the chain of global resilience: disaster caches, prediabetes, Ebola virus disease, and the future of point of care. Point Care. (2015) 15:43–58. 10.1097/POC.0000000000000080

[18] Kost GJ Pratumvinit B Diabetes Spatial Care Paths™, leading edge HbA1c testing, facilitation thresholds, proactive-preemptive strategic intelligence, and unmanned aerial vehicles in limited-resource countries. Point Care. (2017) 16:12–31. 10.1097/POC.0000000000000122

[19] Kost GJ Zadran A Duong TTB Pham TT Ho AVD Nguyen NV Point-of-care diagnosis of acute myocardial infarction in Central Vietnam: international exchange, needs assessment, and Spatial Care Paths™. Point Care. (2018) 17:73–92. 10.1097/POC.0000000000000167 30245595 PMC6135481

[20] Ventura IJ Zadran A Zadran L Duong TB Tang TP Kost GJ Rapid diagnosis and effective monitoring of diabetes mellitus in Central Vietnam: point-of-care needs, improved patient access, and spatial care path integration for enhanced public health. Point Care. (2019) 18:1–8. 10.1097/POC.0000000000000178

[21] Kost GJ Point-of-care Cardiac biomarkers in Vietnam (the Philippines, Taiwan, and Thailand). In: Point of Care in Limited-Resource Settings Symposium, 26th International Molecular Med Tri•Con. San Francisco, CA: Cambridge Healthtech Institute (2019). p. 21.

[22] Grusky O Roberts KJ Swanson AN Rhoades H Lam M. Staff strategies for improving HIV detection using mobile HIV testing. Behav Med. (2010) 35:101–11. 10.1080/08964280903334501 19933057

[23] Goswami ND Hecker E Holland DP Naggle S Cox GM Mosher A. Feasibility and willingness-to-pay for integrated community-based tuberculosis testing. BMC Infect Dis. (2011) 11:305–10. 10.1186/1471-2334-11-305 22047015 PMC3217890

[24] Ferguson WJ Louie RF Tang CS Vy JH Wallace AP Kost GJ Geographic information systems can enhance crisis standards of care during complex emergencies and disasters: a strategy for global positioning system-tracked, H_2_ fuel cell-powered, and knowledge-optimized point-of-care medical intelligence. Point Care. (2012) 11:184–90. 10.1097/POC.0b013e3182666da9

[25] Nagata T Kimura Y Ishii M. Use of a geographic information system (GIS) in the medical response to the Fukushima nuclear disaster in Japan. Prehosp Disaster Med. (2012) 27:213–5. 10.1017/S1049023X1200060X 22587878

[26] Alegana VA Atkinson PM Wright JA Kamwi R Uusiku P Katokele S. Estimation of malaria incidence in northern Namibia in 2009 using Bayesian conditional-autoregressive spatial-temporal models. Spatial Spatio-temporal Epidemiol. (2013) 7:25–36. 10.1016/j.sste.2013.09.001 24238079 PMC3839406

[27] Yao J Agadjanian V Murray AT. Spatial and social inequities in HIV testing utilization in the context of rapid scale-up of HIV/AIDS services in rural Mozambique. Health Place. (2014) 28:133–41. 10.1016/j.healthplace.2014.04.007 24835024 PMC4609644

[28] Ferguson WJ Louie RF Katip P Kost GJ Chapter 35: use of geographic information systems for placement and management of point-of-care technologies in small-world networks. In: Kost GJ Curtis CM editors. Global Point of Care: Strategies for Disasters, Emergencies, and Public Health Preparedness. Washington DC: AACC Press-Elsevier (2015). p. 393–404.

[29] Ferguson W Kost G Streamlining Health Access Through Point of Care Technologies: A Spatial Model. Davis, CA: Point-of-Care Testing Center for Teaching and Research (2015).

[30] Ferguson WJ Kemp K Kost G. Using a geographic information system to enhance patient access to point-of-care diagnostics in a limited-resource setting. Int J Health Geogr. (2016) 15:1–12. 10.1186/s12942-016-0037-9 26932155 PMC4774034

[31] Larocca A Visconti RM Marconi M Malaria diagnosis and mapping with m-Health and geographic information systems (GIS): evidence from Uganda. Malar J. (2016) 15:520:1–12. 10.1186/s12936-016-1546-5 PMC5075756 27776516

[32] Lin JC Kost GJ Ferguson W Bio-innovation in Taiwan, the first survey of point-of-care professional needs, and geospatially enhanced resilience in at-risk settings. Point Care. (2017) 16:78–88. 10.1097/POC.0000000000000134

[33] Kuupiel D Adu KM Apiribu F Bawontuo V Adogboba DA Ali KT Geographic accessibility to public health facilities providing tuberculosis testing services at point-of-care in the upper east region, Ghana. BMC Public Health. (2019) 19:718:1–12. 10.1186/s12889-019-7052-2 PMC6558903 31182068

[34] Hill CE Burd EM Kraft CS Ryan EL Duncan A Winkler AM. Laboratory test support for Ebola patients within a high-containment facility. Lab Med. (2014) 45:e109–11. 10.1309/LMTMW3VVN20HIFS 25184220

[35] Kost GJ Ferguson WJ Truong A-T Prom D Hoe J Banpavichit A The Ebola Spatial Care Path™: point-of-care lessons learned for stopping outbreaks. Clin Lab Int. (2015) 39:6–14.

[36] Kost GJ Ferguson W Truong A-T Hoe J Prom D Banpavichit A. Molecular detection and point-of-care testing in Ebola virus disease and other threats: a new global public health framework to stop outbreaks. Expert Rev Mol Diagnostics. (2015) 15:1245–59. 10.1586/14737159.2015.1079776 26367243 PMC7103715

[37] Shorten RJ Brown CS Jacobs M Rattenbury S Simpson AJ Mepham S. Diagnostics in Ebola virus disease in resource-rich and resource-limited settings. PLoS Negl Trop Dis. (2016) 10:1–16. 10.1371/journal.pntd.0004948 27788135 PMC5082928

[38] Boonlert W Kost GJ Jiraviriyakul A Tangvarasittichai S Point-of-care testing on a mobile medical unit in northern Thailand: screening for hyperglycemia, hyperlipidemia, and thalassemia trait. Point Care. (2006) 5:164–7. 10.1097/01.poc.0000232581.36762.21

[39] Diers J Kouriba B Ladan Fofana L Fleischmann E Starke M Diallo S. Mobile laboratories for rapid deployment and their contribution to the containment of emerging diseases in Sub-Saharan Africa, illustrated by the example of Ebola virus disease. Med Sante Trop. (2015) 25:229–33. 10.1684/mst.2015.0485 26446739

[40] Mansuy JM. Mobile laboratories for Ebola and other pathogens. Lancet Infect Dis. (2015) 15:1135. 10.1016/S1473-3099(15)00309-6 26461946

[41] de La Vega MA Bello A Chaillet P Kobinger GP. Diagnosis and management of Ebola samples in the laboratory. Expert Rev Anti Infect Ther. (2016) 14:557–67. 10.1080/14787210.2016.1176912 27176909

[42] Racine T Kobinger GP Challenges and perspectives on the use of mobile laboratories during outbreaks and their use for vaccine evaluation. Hum Vaccin Immunother. (2019) 20:1–5. 10.1080/21645515.2019.1597595 PMC6816390 30893007

[43] Kost GJ Sakaguchi A Curtis C Tran NK Katip P Louie RF. Enhancing standards of care using innovative point-of-care testing. Am J Disaster Med. (2011) 6:351–68. 10.5055/ajdm.2011.0074 22338316 PMC3434883

[44] Kost GJ Chapter 24: point-of-care testing for Ebola and other highly infectious threats: principles, practice, and strategies for stopping outbreaks. In: Shephard M editor. A Practical Guide to Global Point-of-care Testing. Canberra: CSIRO (Commonwealth Scientific and Industrial Research Organization) (2016). p. 291–305.

[45] Herr DM Newton NC Santrach PJ Hankins DG Burritt MF. Airborne and rescue point-of-care testing. Am J Clin Pathol. (1995) 104 (4 Suppl. 1):S54–8. 7484950

[46] Davey AL Macnab AJ Green G. Changes in pCO_2_ during air medical transport of children with closed head injuries. Air Med J. (2001) 20:27–30. 10.1016/S1067-991X(01)70043-0 11438810

[47] Di Serio F Petronelli MA Sammartino E. Laboratory testing during critical care transport: point-of-care testing in air ambulances. Clin Chem Lab Med. (2010) 48:955–61. 10.1515/CCLM.2010.190 20406127

[48] Louie RF Ferguson WJ Curtis CM Vy JH Tang CS Kost GJ. Effects of environmental conditions on point-of-care cardiac biomarker test performance during a simulated rescue: implications for emergency and disaster response. Am J Disaster Med. (2013) 8:205–12. 10.5055/ajdm.2013.0126 24352994

[49] Tideman PA Tirimacco R Senior DP Setchell JJ Huynh LT Tavella R. Impact of a regionalised clinical cardiac support network on mortality among rural patients with myocardial infarction. Med J Aust. (2014) 200:157–60. 10.5694/mja13.10645 24528431

[50] Sorensen JT Stengaard C Chapter 50: prehospital application of cardiac biomarkers for decision support of patients with suspected acute myocardial infarction. In: Kost GJ Curtis CM editors. Global Point of Care: Strategies for Disasters, Emergencies, and Public Health Preparedness. Washington, DC: AACC Press-Elsevier (2015). p. 569–76.

[51] Rasmussen MB Stengaard C Sørensen JT Riddervold IS Hansen TM Giebner M. Predictive value of routine point-of-care cardiac troponin T measurement for prehospital diagnosis and risk-stratification in patients with suspected acute myocardial infarction. Eur Heart J Acute Cardiovasc Care. (2017) 8:299–308. 10.1177/2048872617745893 29199427

[52] Kost GJ How POCT Improves Care and Educates Physicians: Exciting Contemporary Examples and Innovative Opportunities, including Point-of-Careology on Mars. In: 12th Annual Convention. Manila: Philippines Council for Quality Assurance in Clinical Laboratories (2015).

[53] Canadian Consortium Bio-Analyzer: Near Real-time Biomedical Results from Space to Earth. (2018). Available online at: http://asc-csa.gc.ca/eng/iss/bio-analyzer.asp

[54] Roda A Mirasoli M Guardigli M Zangheri M Caliceti C Calabria D. Advanced biosensors for monitoring astronauts' health during long-duration space missions. Biosens Bioelectron. (2018) 111:18–26. 10.1016/j.bios.2018.03.062 29631159

[55] Zangheri M Mirasoli M Guardigli M Di Nardo F Anfossi L Baggiani C. Chemiluminescence-based biosensor for monitoring astronauts' health status during space missions: results from the International Space Station. Biosens Bioelectron. (2019) 129:260–68. 10.1016/j.bios.2018.09.059 30292340

[56] Kamanga A Moono P Stresman G Mharakurwa S Shiff C Rural health centers, communities and malaria case detection in Zambia using mobile telephones: a means to detect potential reservoirs of infection in unstable transmission conditions. Malar J. (2010) 9:96 10.1186/1475-2875-9-96 20398318 PMC2861692

[57] Laksanasopin T Gup TW Sia SK Chapter 36: integrating diagnostics tests and connectivity to enable disease diagnosis and tracking in remote settings. In: Kost GJ Curtis CM editors. Global Point of Care: Strategies for Disasters, Emergencies, and Public Health Preparedness. Washington, DC: AACC Press-Elsevier (2015). p. 405–10.

[58] Smith S Oberholzer A Korvink JG Mager D Land K. Wireless colorimetric readout to enable resource-limited point-of-care. Lab Chip. (2019) 19:3344–53. 10.1039/C9LC00552H 31502631

[59] Amukele TK Street J Carroll K Miller H Zhang SX. Drone transport of microbes in blood and sputum laboratory specimens. J Clin Microbiol. (2016) 54:2622–5. 10.1128/JCM.01204-16 27535683 PMC5035434

[60] Amukele TK Sokoll LJ Pepper D Howard DP Street J. Can unmanned aerial systems (drones) be used for the routine transport of chemistry, hematology, and coagulation laboratory specimens? PLoS ONE. (2015) 10:e0134020. 10.1371/journal.pone.0134020 26222261 PMC4519103

[61] Amukele TK Hernandez J Snozek CLH Wyatt RG Douglas M Amini R. Drone transport of chemistry and hematology samples over long distances. Am J Clin Pathol. (2017) 148:427–35. 10.1093/ajcp/aqx090 29016811

[62] Amukele T. Current state of drones in healthcare: challenges and Opportunities. J Appl Lab Med. (2019) 4:296–8. 10.1373/jalm.2019.030106 31639681

[63] Priye A Wong S Bi Y Carpio M Chang J Coen M Lab on a drone: toward pinpoint deployment of smartphone-enabled nucleic acid-based diagnostic for mobile healthcare. Anal Chem. (2016) 88:4651–60. 10.1021/acs.analchem.5b04153 26898247 PMC4857158

[64] Kost GJ Peungposop N Kulrattanamaneeporn C Wongboonsin K Charuruks N Surasiengsunk S Chapter 3: minimizing health problems to optimize the demographic dividend: the role of point-of-care testing. In: Wongboonsin K Guest P editors. The Demographic Dividend: Policy and Options for Asia. Chulalongkorn: Chulalongkorn University Printing House (2005). p. 56–89.

[65] Kost GJ Katip P Kanokslip A Mecozzi DM A new demographic strategy for point-of-need medical testing: linking health resource scores, poverty levels, and care paths. J Demography. (2011) 27:1–31.

[66] Kost GJ Katip P Vinitwatanakhun C Diagnostic testing strategies for health care delivery during the Great Bangkok Flood and other weather disasters. Point Care. (2012) 11:191–9. 10.1097/POC.0b013e318265f255

[67] Kost GJ Katip P Curtis C. Strategic point-of-care requirements of hospitals and public health for preparedness in regions at risk. Point Care. (2012) 11:114–8. 10.1097/POC.0b013e31825a2442 23049470 PMC3462019

[68] Kost GJ Katip P Kulrattanamaneeporn S Gentile N Point-of-care testing value proposition for disaster preparedness in small-world networks: post-Tsunami Phang Nga Province, Coastal Thailand. Pont Care. (2013) 12:9–22. 10.1097/POC.0b013e318265f3d4

[69] Pigott DM Golding N Mylne A Huang Z Henry AJ Weiss DJ. Mapping the zoonotic niche of Ebola virus disease in Africa. eLife. (2014) 3:e04395. 10.7554/eLife.04395 25201877 PMC4166725

[70] Pigott DM Millear AI Earl L Morozoff C Han BA Shearer FM Updates to the zoonotic niche map of Ebola virus disease in Africa. eLife. (2016) 45:e16412 10.7554/eLife.16412 PMC4945152 27414263

[71] Baizurah MH The Point of Care Testing Steering Committee National Point of Care Testing Policy and Guidelines. Putrajaya: Ministry of Health Malaysia Medical Development Division (2012). Available online at: http://www.moh.gov.my/images/gallery/Garispanduan/National_Point_of_Care_Testing.pdf

[72] Kost GJ Baizurah MH Chapter 53: national point of care testing policy and guidelines in Malaysia, standards of care, and impact worldwide. In: Kost GJ Curtis CM editors. Global Point of Care: Strategies for Disasters, Emergencies, and Public Health Preparedness. Washington, DC: AACC Press-Elsevier (2015). p. 595–610.

[73] Kost GJ. The hybrid laboratory: shifting the focus to the point of care. Med Lab Obs. (1992) 24 (Suppl. 9):17–21. 10171231

[74] Kost GJ. The hybrid laboratory. The clinical laboratory of the 1990s is a synthesis of the old and the new. Arch Pathol Lab Med. (1992) 116:1002–3. 1417438

[75] Kost GJ. New whole blood analyzers and their impact on cardiac and critical care. Crit Rev Clin Lab Sci. (1993) 30:153–202. 10.3109/10408369309084667 8363743

[76] Kost GJeditors. chapter 2: The hybrid laboratory, therapeutic turnaround time, critical limits, performance maps, and Knowledge Optimization^®^. In: Principles and Practice of Point-of-Care Testing. Philadelphia, PA: Lippincott Williams and Wilkins (2002). p. 13–25.

[77] Liu X Zhu X Kost GJ Liu J Huang J Liu X The creation of Point-of-Careology. Point Care. (2019) 18:77–84. 10.1097/POC.0000000000000191

[78] Kost GJ. Principles and Practice of Point-of-Care Testing. Philadelphia, PA: Lippincott Williams and Wilkins (2002).

[79] Kost GJ Curtis CM Global Point of Care: Strategies for Disasters, Emergencies, and Public Health Preparedness. Washington, DC: AACC Press-Elsevier (2015).

[80] Kost GJ Ehrmeyer SS Chernow B Winkelman JW Zaloga GP Dellinger RP. The laboratory-clinical interface: point-of-care testing. Chest. (1999) 115:1140–54. 10.1378/chest.115.4.1140 10208220

[81] Bricker D Ibbitson J Empty Planet: The Shock of Global Population Decline. Danvers, MA: Crown (2019).

[82] Global Preparedness Monitoring Board A World at Risk: Annual Report on Global Preparedness for Health Emergencies. Geneva: World Health Organization (2019).

[83] Kost GJ. Molecular and point-of-care diagnostics for Ebola and new threats: national POCT policy and guidelines will stop epidemics. Expert Rev Mol Diagn. (2018) 18:657–73. 10.1080/14737159.2018.1491793 29933717

[84] Gleason BL Foster S Wilt GE Miles B Lewis B Cauthen K. Geospatial analysis of household spread of Ebola virus in a quarantined village - Sierra Leone, 2014. Epidemiol Infect. (2017) 145:2921–29. 10.1017/S0950268817001856 28826426 PMC7315611

[85] Gomez-Barroso D Velasco E Varela C Leon I Cano R. Spread of Ebola virus disease based on the density of roads in West Africa. Geospat Health. (2017) 12:552. 10.4081/gh.2017.552 29239551

[86] Gonzalez JP Souris M Valdivia-Granda W. Global spread of hemorrhagic fever viruses: predicting pandemics. Methods Mol Biol. (2018) 1604:3–31. 10.1007/978-1-4939-6981-4_1 28986822 PMC7120037

[87] Lau MSY Gibson GJ Adrakey H McClelland A Riley S Zelner J A mechanistic spatio-temporal framework for modeling individual-to-individual transmission-With an application to the 2014–2015 West Africa Ebola outbreak. PLoS Comput Biol. (2017) 13:e1005798 10.1371/journal.pcbi.1005798 29084216 PMC5679647

[88] Kost GJ Zadran A Zadran L Ventura I. Point-of-care testing curriculum and accreditation for Public Health—Enabling preparedness, response, and higher standards of care at points of need. Front Public Health. (2019) 6:1–15. 10.3389/fpubh.2018.00385 30761282 PMC6361824

[89] Kost GJ Zadran A. Schools or public health should be accredited for, and teach the principles and practice of point-of-care testing. J Appl Lab Med. (2019) 4:278–83. 10.1373/jalm.2019.029249 31639676

[90] Burnham CD Kwon JH Burd EM Campbell S Iwen PC Miller MB. Are we there yet? Laboratory preparedness for emerging infectious diseases. Clin Chem. (2017) 63:807–11. 10.1373/clinchem.2016.265850 28115392 PMC7108463

[91] Fuzery AK Bobyak J Chang E Shaman R Venner AA. Challenges of point-of-care testing in ambulances. J Appl Lab Med. (2019) 4:293–5. 10.1373/jalm.2019.029439 31639680

[92] Phipps WS Yen Z Bae C Sharpe JZ Bishara AM Nelson ES Reduced-gravity environment hardware demonstrations of a prototype miniaturized flow cytometer and companion microfluidic mixing technology. J Visualized Experiments. (2014) 93:e51743 10.3791/51743 PMC4354048 25490614

[93] Wang P Kricka LJ. Current and emerging trends in point-of-care technology and strategies for clinical validation and implementation. Clin Chem. (2018) 64:1439–52. 10.1373/clinchem.2018.287052 29884677

[94] Wu A “On vivo” and wearable clinical laboratory (sic) testing devices for emergency and critical care testing. J Appl Lab Med. (2019) 4:254–63. 10.1373/jalm.2018.028654 31639672

[95] Cummins BM Ligler FS Walker GM. Point-of care diagnostics for niche applications. Biotechnol Adv. (2016) 34:161–76. 10.1016/j.biotechadv.2016.01.005 26837054 PMC4833668

[96] Kost GJ Chapter 1: overview of point-of-care testing: goals, guidelines, and principles. In: Charuruks N Manarom W editors. Point of Care Testing for Thailand. Bangkok: Thai Publishers (2006). p. 1–28.

[97] Kost GJ Chapter 10: point-of-care testing in province hospitals and primary care units (PCUs): optimizing critical care and disaster response. In: Charuruks N Manarom W editors. Point of Care Testing for Thailand. Bangkok: Thai Publishers (2006). p. 159–77.

[98] Wattanawaitunechai C Peacock SJ Jitpratoom P. Tsunami in Thailand—Disaster management in a district hospital. NEJM. (2005) 352:362–4. 10.1056/NEJMp058040 15758004

[99] VanRooyen M Leaning J. After the Tsunami—Facing the public health challenges. NEJM. (2005) 352:435–8. 10.1056/NEJMp058013 15689579

[100] Bronisch T Maragkos M Freyer C Müller-Cyran A Butollo W Weimbs R. Crisis intervention after the Tsunami in Phuket and Khao Lak. Crisis. (2006) 27:42–7. 10.1027/0227-5910.27.1.42 16642915

[101] Kost GJ Future Role of the Laboratory in Emergency Medicine. New York, NY: The Laboratory's Role in Emergency Medicine (Natural Disasters), The Institute for Continuing Education of the 1199SEIU League Training and Upgrading Fund, 1199SEIU United Healthcare Workers East Professional and Technical Department, Gerald W. Lynch Theater at John Jay College (2013).

[102] Thirsk R. Physicians as astronauts. McGill Med J. (2011) 13:69–75. 22363198 PMC3277415

[103] World Health Organization Second WHO Model List of Essential In Vitro Diagnostics. Geneva: WHO (2019).

[104] Zadran A Kost GJ Enabling rapid intervention and isolation for patients with highly infectious diseases at points of need. J Hosp Mgt Health Policy. (2019) 4:1–7. 10.21037/jhmhp.2018.12.04

[105] Kost GJ Preparedness, Point-of-care Testing, and Global Resilience: Stopping Outbreaks and Assuring Equitable Care in Africa and Southeast Asia. Davis, CA: Knowledge Optimization® (2017).

[106] Kost GJ Preparedness, Point-of-care Testing, and Resilience—Stopping Outbreaks and Assuring Equitable Care for Island and High Risk Association of Southeast Asian Nations. Davis, CA: Knowledge Optimization® (2019).

[107] Ailworth E Growth fuels size of catastrophes. Wall Street J. (2019) 273:A3.

[108] Katz R Banaski JA Essentials of Public Health Preparedness and Management. Burlington, MA: Jones and Bartlett Learning (2019).

[109] McKinney S Papke ME Public Health Emergency Preparedness: A Practical Approach for the Real World. Burlington, MA: Jones and Bartlett Learning (2019).

[110] Carroll LN Au AP Detwiler LT Fu T-C Painter IS Abernethy NF Visualization and analytic tools for infectious disease epidemiology: a systematic review. J Biomed Info. (2014) 51:287–98. 10.1016/j.jbi.2014.04.006 PMC5734643 24747356

[111] Ramadan AAB Jackson-Thompson J Boren SA. Geographic information systems: usability, perception, and preferences of public health professionals. Online J Public Health Informatics. (2017) 9:e191. 10.5210/ojphi.v9i2.7437 29026456 PMC5630278

[112] Leligdowicz A Fischer WA Uyeki TM Fletcher TE Adhikari NK Portella G. Ebola virus disease and critical illness. Crit Care. (2016) 20:217. 10.1186/s13054-016-1325-2 27468829 PMC4965892

[113] Dean CL Hill CE. Caring for patients with Ebola virus disease: are U.S. biocontainment centers ready for the next outbreak? Semin Diagn Pathol. (2019) 36:160–3. 10.1053/j.semdp.2019.04.007 31010606

[114] Vashist SK. Point-of-care diagnostics: recent advances and trends. Biosensors. (2017) 7:1–4. 10.3390/bios7040062 29258285 PMC5746785

